# Supplementary material for: The Intestine Harbors Functionally Distinct Homeostatic Tissue-Resident and Inflammatory Th17 Cells
Source: Immunity. 2019 Jul 16;51(1):77–89.e6. doi: 10.1016/j.immuni.2019.05.004 (PMC6642154; doi:10.1016/j.immuni.2019.05.004)
Supplement: Document S1. Figures S1–S5 [file mmc1.pdf]

Immunity, Volume *51*

Supplemental Information

The Intestine Harbors Functionally Distinct Homeostatic Tissue-Resident and Inflammatory Th17 Cells

Sara Omenetti, Claudio Bussi, Amina Metidji, Andrea Iseppon, Sunjae Lee, Mauro Tolaini, Ying Li, Gavin Kelly, Probir Chakravarty, Saeed Shoaie, Maximiliano G. Gutierrez, and Brigitta Stockinger

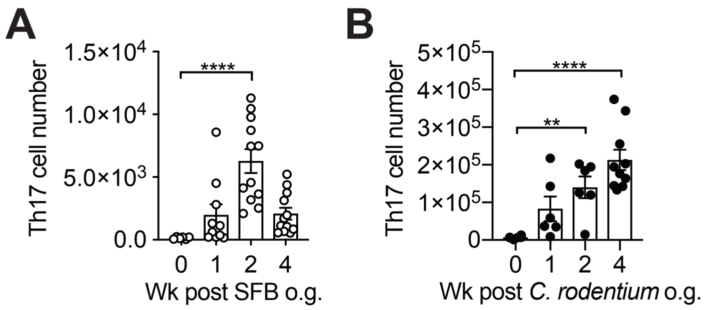

Figure S1. Th17 cell induction by SFB in the colon and C. rodentium in the small intestine, related to Figure 1.

Absolute numbers of Th17 cells in the colon of mice colonised with SFB (A) and the small intestine of mice infected with C. rodentium (B) at 0 (n=6), 1 (n=10-6), 2 (n=12-6) and 4 (n=12-10) weeks post-gavage. In the graphs, bars show the mean and each symbol represent an individual mouse from two pooled independent experiments. **p < 0.01, ****p < 0.0001 by one-way ANOVA with Dunnett's post-test.

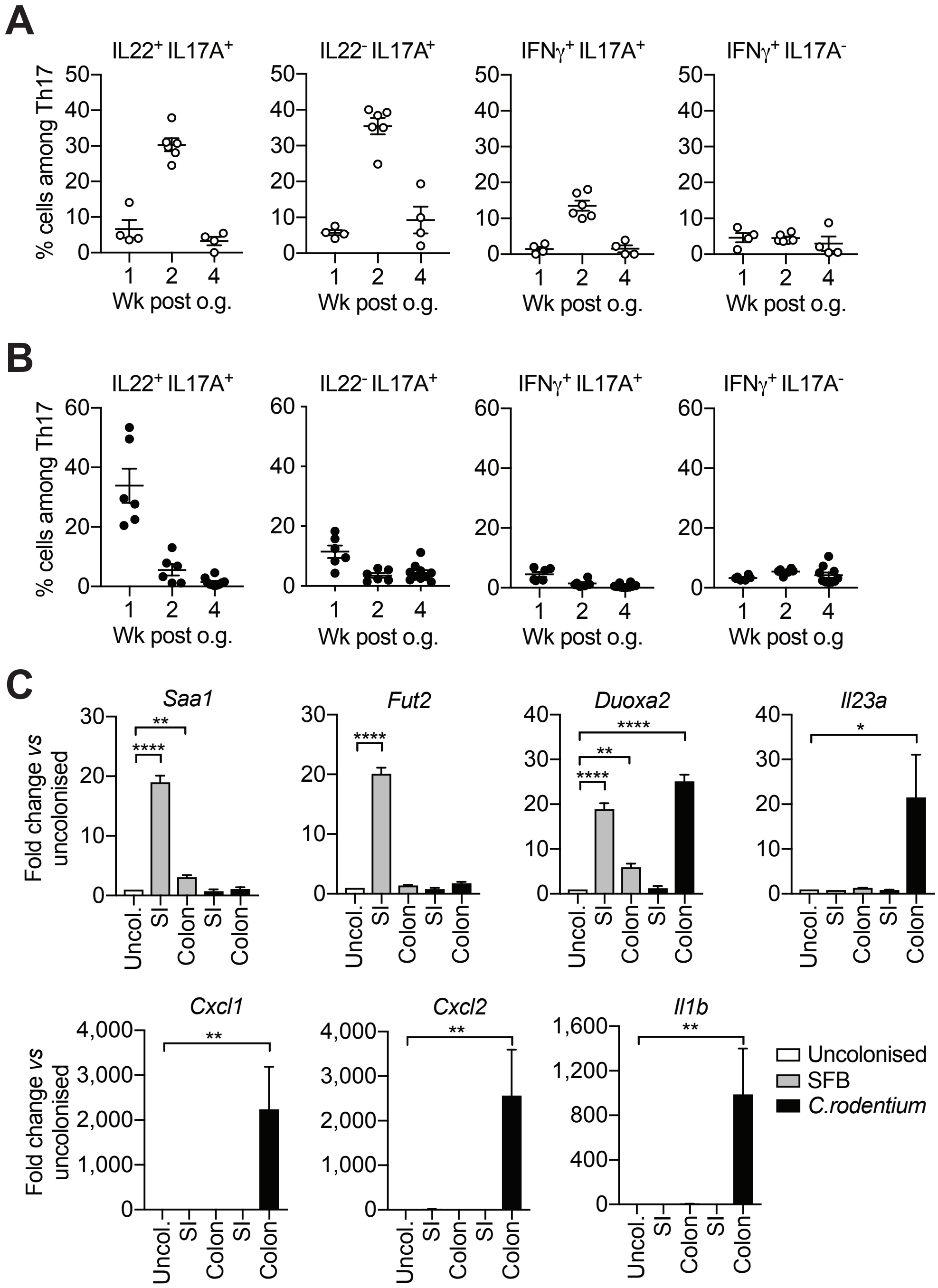

Figure S2. Cytokine production by SFB-elicited Th17 cells in the colon, related to Figure 2. Quantification of intracellular staining for IL-17A and IL-22 or IFN-γ in Th17 cells from the colon of mice colonised with SFB (A) and the small intestine of mice infected with C. rodentium (B) at 1 (n=4 and 6), 2 (n=6) and 4 (n=4 and 10) weeks post-gavage. Lamina propria cells were isolated at the indicated time-point, re-stimulated with PMA and Ionomycin and Brefeldin A for 2 hours and analysed by FACS. Bars show the mean ± s.e.m. for the indicated populations and each symbol represents an individual mouse from two pooled independent experiments. (C) Fold change induction of listed genes quantified by qPCR in the SI and colon of mice colonised with SFB (n=3 and n=4) or infected with C. rodentium (n=5), 1 week post-gavage. Fold change is calculated on the matching uncolonised organ (uncol.) (n=6) and gene expression normalised against ß-2-microglobulin. Bars show the mean + s.e.m. from two pooled independent experiments. *$p < 0.05$, **$p < 0.01$, ****$p < 0.0001$ by one-way ANOVA with Dunnett’s post-test.

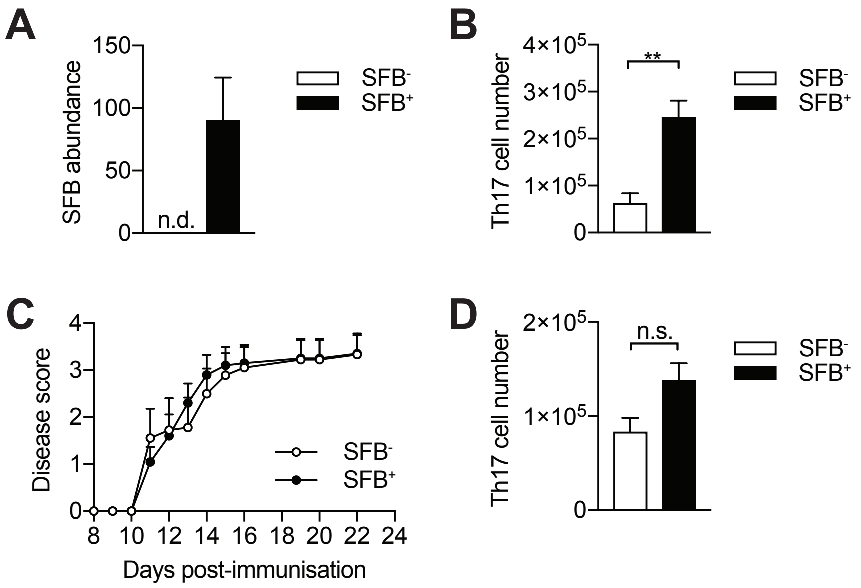

Figure S3. SFB does not impact the course of EAE, related to Figure 3.
(A) Relative abundance of Segmented Filamentous bacteria (SFB) in the faeces of mice reconstituted with SFB^- (n=4) or SFB^+ (n=6) faeces at 12 days upon EAE immunisation. SFB genomic 16s was quantified in the faeces by qPCR analysis. Abundance of SFB was normalised to Eubacteria. (B) Absolute numbers of Th17 cells in the small intestine (B) or spinal cord (D) of SFB^- and SFB^+ mice (n=4) at 12 days upon EAE immunisation. (C) Clinical scores of SFB^- (n=10) and SFB^+ mice (n=10) mice upon EAE immunisation. Results are pooled from two independent experiments. Data are shown as mean + s.e.m. **$p < 0.01$ by Student t-test. n.s., not significant; n.d., not detected.

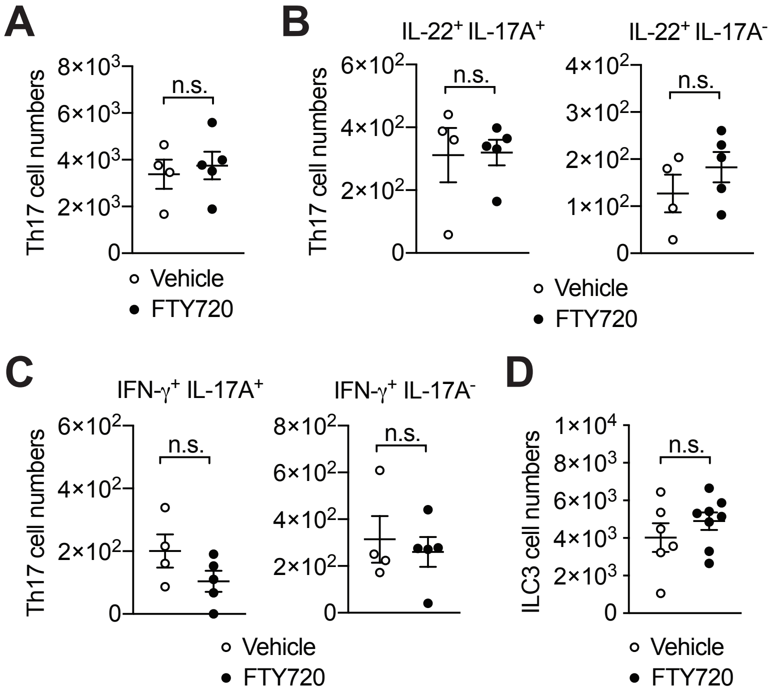

Figure S4. FTY720 treatment does not affect numbers of SFB-elicited Th17 cells and C. rodentium-elicited ILC3 cells, related to Figure 4.
Mice were orally gavaged with SFB+ faeces 4 weeks prior the gavage with PBS (A, B, C) or C.rodentium (D) (at day 0). Six days prior the PBS or C.rodentium gavage, mice were intra-peritoneally injected with FTY720 (3mg/kg) or control vehicle every day. FTY720 and control vehicle administration continued after the PBS or C. rodentium gavage every other day until the end of the experiment. (A) Absolute numbers of colonic Th17 cells (n=4 and n=5). Quantification of intracellular staining in colonic Th17 cells (n=4 and n=5) for IL-17A and IL-22 (B) or IFN-γ (C). (D) Absolute numbers of colonic ILC3 cells (n=6 and n=8). Bars show mean ± s.e.m. and each symbol represent an individual mouse. Results are representative of two independent experiments. n.s., not significant by Student t-test.

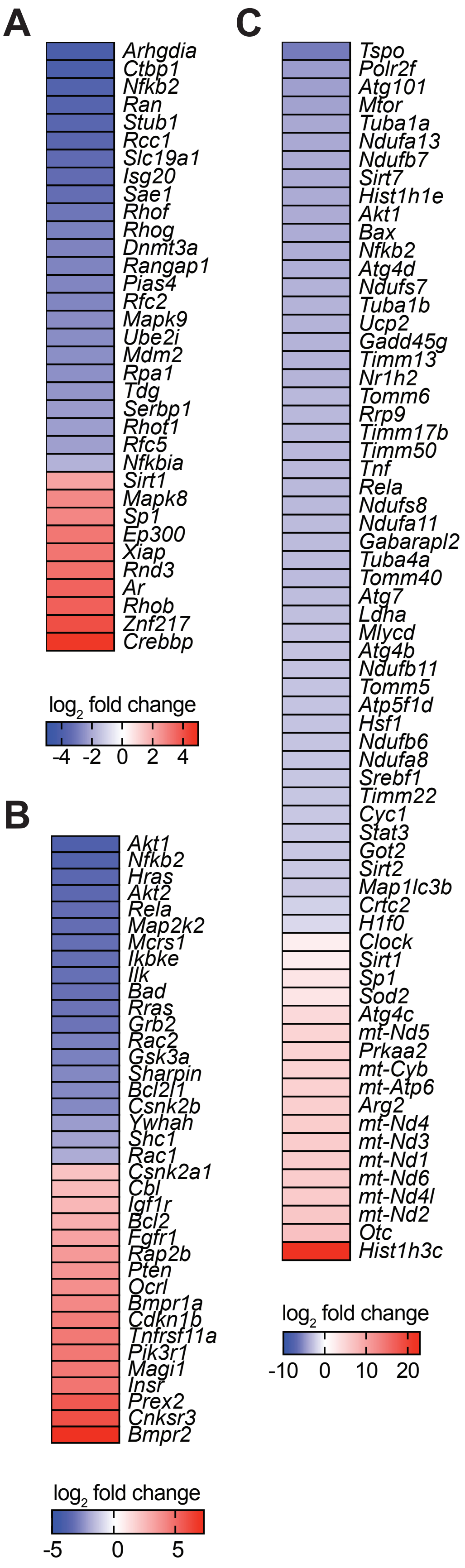

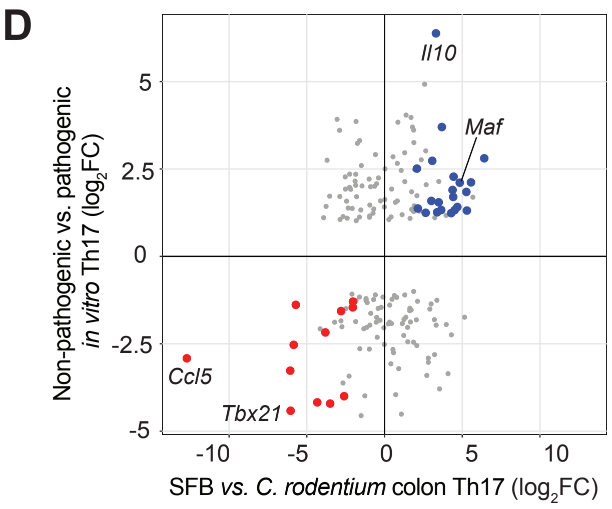

E

	SFB *vs. C.rodentium* colonic Th17	Non-pathogenic *vs.* pathogenic Th17
Lgr4	6.4	2.8
Nhsl1	5.6	2.1
Ptgfrn	5.3	1.3
Dfna5	5.3	1.8
Maf	4.8	2.1
Sgce	4.7	1.4
Pik3r1	4.5	1.3
Acvr2a	4.4	2.3
Alpk2	4.4	1.7
Sgip1	4.4	1.9
Mob3b	4.3	1.2
Gcnt1	3.7	3.7
Sfmbt2	3.6	1.3
Trio	3.5	1.5
Dcbld2	3.4	1.3
Il10	3.3	6.4
Prnp	3.1	2.7
Tmigd1	3.0	1.6
Skil	2.6	1.2
Pitpnc1	2.1	1.4
Acpp	2.1	2.5

F

	SFB *vs. C.rodentium* colonic Th17	Non-pathogenic *vs.* pathogenic Th17
Ccl5	-12.7	-2.9
Ifitm1	-6.1	-3.3
Tbx21	-6.0	-4.4
Ermn	-5.8	-2.5
Sell	-5.7	-1.4
Nkg7	-4.3	-4.2
Serpine2	-3.8	-2.2
Ifitm3	-3.5	-4.2
Lrmp	-2.8	-1.6
Cxcr3	-2.6	-4.0
Mbnl3	-2.0	-1.5
Uba7	-2.0	-1.3

Figure S5. Gene expression of pathways activated in SFB-induced colonic Th17 cells and shared gene expression with in vitro generated Th17 cells, related to Figure 5.
Expression (log2-fold change) of genes identified by ingenuity pathway analysis (IPA) in sumoylation (A), PTEN (B) and sirtuin (C) pathways in colonic SFB- vs. C.rodentium-induced Th17 cells. (D) Scatter plot of genes identified from in vitro generated non-pathogenic vs. pathogenic Th17 cells (Ghoreschi et al, 2010) ($p < 0.001$) detected in SFB or C. rodentium colon Th17 cells. Genes in blue (listed in E) and in red (listed in F) are significantly different in SFB vs. C. rodentium colon Th17 cells (adj $p < 0.05$) and follow the same direction of the published dataset. Values in E and F are $\log_2$ fold change of genes in the indicated comparison.
