# Supplementary material for: The Intestine Harbors Functionally Distinct Homeostatic Tissue-Resident and Inflammatory Th17 Cells
Source: Immunity. 2019 Jul 16;51(1):77–89.e6. doi: 10.1016/j.immuni.2019.05.004 (PMC6642154; doi:10.1016/j.immuni.2019.05.004)
Supplement: Document S2. Article plus Supplemental Information [file mmc3.pdf]

Immunity

Article

The Intestine Harbors Functionally Distinct Homeostatic Tissue-Resident and Inflammatory Th17 Cells

Graphical Abstract

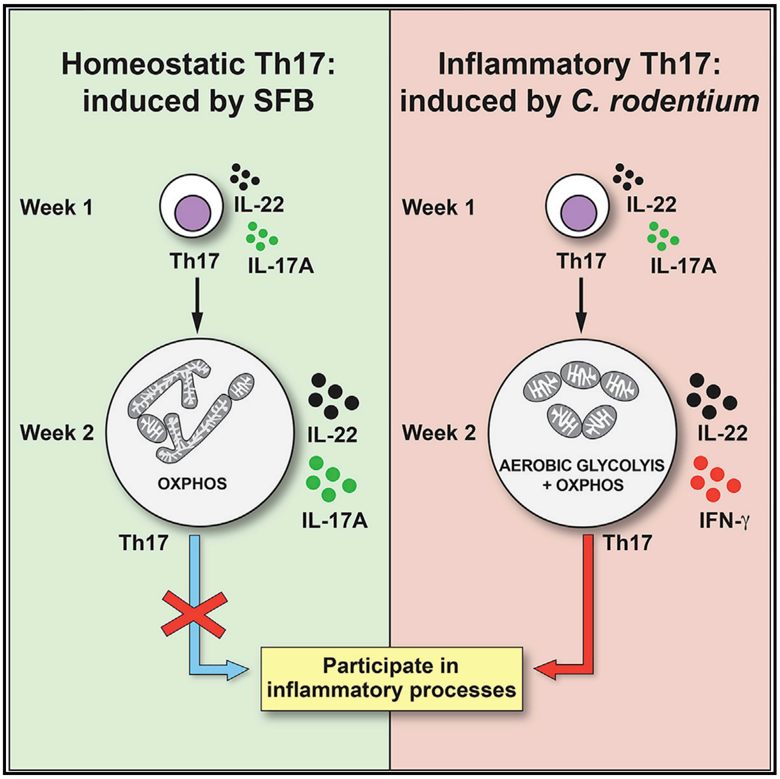

Authors

Sara Omenetti, Claudio Bussi, Amina Metidji, ..., Saeed Shoaie, Maximiliano G. Gutierrez, Brigitta Stockinger

Correspondence

brigitta.stockinger@crick.ac.uk

In Brief

The distinctions between inflammatory Th17 cells elicited by pathogens and tissue-resident homeostatic Th17 cells elicited by commensals are unclear. Omenetti et al. show that tissue-resident Th17 cells, in contrast to pathogen-elicited Th17 cells, exhibit little plasticity towards inflammatory cytokines, show muted metabolism, and do not participate in inflammatory reactions. These findings highlight the link between metabolic fitness and functional state.

Highlights

- Tissue-resident, SFB-elicited Th17 cells are non-inflammatory
- *Citrobacter*-elicited Th17 cells show high plasticity towards inflammatory cytokines
- SFB Th17 cells are metabolically similar to resting memory cells
- *Citrobacter* Th17 cells are highly glycolytic effector cells

Omenetti et al., 2019, Immunity *51*, 77–89
July 16, 2019 © 2019 The Author(s). Published by Elsevier Inc.
https://doi.org/10.1016/j.immuni.2019.05.004

Immunity
Article

CellPress

The Intestine Harbors Functionally Distinct Homeostatic Tissue-Resident and Inflammatory Th17 Cells

Sara Omenetti,[1] Claudio Bussi,[1] Amina Metidji,[1,3] Andrea Iseppon,[1] Sunjae Lee,[1,2] Mauro Tolaini,[1] Ying Li,[1] Gavin Kelly,[1] Probir Chakravarty,[1] Saeed Shoaie,[1,2] Maximiliano G. Gutierrez,[1] and Brigitta Stockinger[1,4,*]
[1]The Francis Crick Institute, 1 Midland Road London NW1 1AT, UK
[2]Centre for Host-Microbiome Interactions, Faculty of Dentistry, Oral & Craniofacial Sciences, King's College London, SE1 9RT, UK
[3]Present Address: Centre de Recherche scientifique et technique en Analyses Physico-Chimiques (C.R.A.P.C), Alger, Algérie
[4]Lead Contact
*Correspondence: brigitta.stockinger@crick.ac.uk
https://doi.org/10.1016/j.immuni.2019.05.004

SUMMARY

T helper 17 (Th17) cells are pathogenic in many inflammatory diseases, but also support the integrity of the intestinal barrier in a non-inflammatory manner. It is unclear what distinguishes inflammatory Th17 cells elicited by pathogens and tissue-resident homeostatic Th17 cells elicited by commensals. Here, we compared the characteristics of Th17 cells differentiating in response to commensal bacteria (SFB) to those differentiating in response to a pathogen (*Citrobacter rodentium*). Homeostatic Th17 cells exhibited little plasticity towards expression of inflammatory cytokines, were characterized by a metabolism typical of quiescent or memory T cells, and did not participate in inflammatory processes. In contrast, infection-induced Th17 cells showed extensive plasticity towards pro-inflammatory cytokines, disseminated widely into the periphery, and engaged aerobic glycolysis in addition to oxidative phosphorylation typical for inflammatory effector cells. These findings will help ensure that future therapies directed against inflammatory Th17 cells do not inadvertently damage the resident gut population.

INTRODUCTION

T helper 17 (Th17) cells are intensely studied because of their known role in the pathogenicity of a variety of inflammatory disorders (Wang et al., 2011; Weaver et al., 2013). However, less well understood are the characteristics of tissue-resident homeostatic intestinal Th17 cells that differentiate in response to the microbiota, notably segmented filamentous bacteria (SFB), and fulfil important roles in protection of the gut barrier (Chung et al., 2012; Lécuyer et al., 2014). To complicate matters further, intestinal Th17 cell responses are also linked with the pathology of inflammatory bowel disease (IBD) (Jostins et al., 2012), and it has been suggested that intestinal Th17 cells contribute to inflammatory disease elsewhere in the body (Lee et al., 2011; Wu et al., 2010).

Substantial efforts have been made to define gene regulatory networks that discriminate pathogenic and non-pathogenic Th17 cells (Gaublomme et al., 2015; Lee et al., 2012). A caveat is that this distinction is so far largely on the basis of Th17 cells generated *in vitro* in the presence of different cytokine cocktails compared with Th17 cells isolated from the inflamed central nervous system (CNS) of mice with active experimental autoimmune encephalomyelitis (EAE). Because Th17 cells are influenced by the local tissue environment they find themselves in and are affected by a multitude of environmental factors, including diet (Endo et al., 2015; Kleinewietfeld et al., 2013; Wu et al., 2013), aryl hydrocarbon receptor (AHR) ligands (Zelante et al., 2013), circadian rhythm (Yu et al., 2013), and the microbiota, it is unlikely that *in-vitro*-differentiated Th17 cells recapitulate the spectrum of biology present in *ex vivo* Th17 cells. Here, we have therefore focused on intestinal Th17 cells either present during homeostasis or induced by infection. This was done by comparing the characteristics of SFB-induced Th17 cells and those of Th17 cells differentiating in response to a pathogen (*Citrobacter rodentium*). Th17 cells induced in response to *C. rodentium* are necessary for clearing the infection, and this results in transient but reversible tissue damage due to their inflammatory properties.

At present, it is unclear what distinguishes inflammatory Th17 cells elicited by pathogens (e.g., *C. rodentium*) and homeostatic tissue-resident Th17 cells elicited by commensals (e.g., SFB). A further consideration is the success in therapeutic targeting of inflammatory Th17 cells in conditions such a psoriasis, or multiple sclerosis via antibodies against interleukin-17A (IL-17A) or IL-12p40 (Patel and Kuchroo, 2015). Such antibodies, on the other hand, proved to worsen the symptoms of Crohn's patients (Hueber et al., 2012). Although reduction of inflammatory cytokine production by Th17 cells does not directly affect the cells themselves, other strategies such as inhibition of retinoic acid-related orphan receptors γt (RORγt) might compromise the maintenance of the homeostatic intestinal Th17 cell population. Transient inhibition of RORγt in a mouse model has been shown to reduce Th17 cell differentiation in response to infection with *C. rodentium*, while sparing group 3 innate lymphoid cells (ILC3s), however the effect on resident small intestinal Th17 cells was not tested (Withers et al., 2016). It is therefore important to understand the intrinsic differences between inflammatory and tissue-resident Th17 cells to ascertain that future therapies

This is an open access article under the CC BY license (http://creativecommons.org/licenses/by/4.0/).

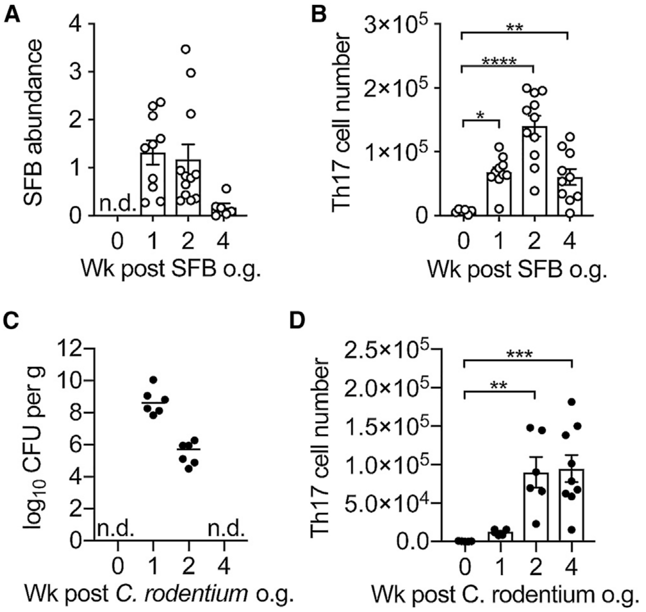

Figure 1. Colonization with SFB and Infection with *C. rodentium* Induce Different Th17 Responses

(A) Relative abundance of SFB in the feces of mice reconstituted with SFB^{+} feces on 0 (n = 10), 1 (n = 10), 2 (n = 12), and 4 (n = 6) weeks after gavage. SFB genomic 16s was quantified in the feces by qPCR analysis. Abundance of SFB was normalized to Eubacteria.

(B and D) Absolute numbers of Th17 cells in the small intestine of mice colonized with SFB (B) and colon of mice infected with *C. rodentium* (D) at 0 (n = 6 and 5), 1 (n = 10 and 6), 2 (n = 11 and 6), and 4 (n = 10 and 9) weeks after gavage.

(C) *C. rodentium* burden in the colon of infected mice at 0 (n = 5), 1 (n = 6), 2 (n = 6), and 4 (n = 9) weeks after gavage.

In the graphs, bars show the mean ± SEM (A, B, and D) or median (C) and each symbol represents an individual mouse from two pooled independent experiments. *p < 0.05, **p < 0.01, ***p < 0.001, ****p < 0.0001 by one-way ANOVA with Dunnett's post-test. n.d., not detected. See also Figure S1.

directed against inflammatory Th17 cells do not inadvertently damage the resident gut population.

Our data show that tissue-resident homeostatic Th17 cells induced by SFB do not participate in inflammatory reactions and exhibit a transcriptional profile indicating downmodulation of inflammatory capacity. In contrast, Th17 cells elicited in response to *C. rodentium* exhibit a high degree of plasticity towards an inflammatory cytokine profile and a transcriptome reflecting inflammatory effector potential. Furthermore, the metabolism of tissue-resident homeostatic Th17 cells resembles more that of resting memory cells, whereas *C.*-rodentium-elicited Th17 cells primarily engage aerobic glycolysis that is commonly associated with inflammatory effector cells. Our findings indicate that there are two functionally distinct Th17 cell populations that might reside in the gut simultaneously.

RESULTS

Segmented Filamentous Bacteria and *Citrobacter rodentium* Induce Qualitatively Different Th17 Responses

SFB are one of the most potent and well-characterized commensal inducers of Th17 cells. Indeed, monocolonization of germ-free mice with SFB promotes a robust Th17 cell response in the small intestinal lamina propria and to a minor extent in the large intestine lamina propria (Ivanov et al., 2009). To further explore Th17 cell induction by SFB, we colonized SFB-negative specific-pathogen free (SPF) mice with SFB by oral gavage with feces from SFB monocolonized germ-free mice. Subsequently, SFB-containing feces were collected from the SFB^{+} mice housed in our SPF colony and used to introduce SFB into experimental mice. To trace the kinetics of Th17 differentiation upon SFB colonization, we introduced SFB into IL-17A fate reporter mice (*Il17a*^{Cre}*R26R*^{eYFP}) by oral gavage, and Th17 cells, permanently marked as eYFP^{+} cells, as well as SFB colonization were then monitored 1, 2, and 4 weeks later. SFB abundance peaked in the feces of colonized mice one week after introduction and then diminished over time (Figure 1A). Although SFB abundance was lower at week 4, it remained detectable at any later time-point, in accordance with other reports showing that SFB is contained after introduction into wild-type animals, but never completely cleared (Kumar et al., 2016). The expansion and containment of SFB correlated with the induction and contraction of Th17 cell numbers in the small intestinal lamina propria (Figure 1B), confirming that Th17 cells are induced upon SFB expansion and, in turn, contribute to control SFB load. Although SFB is confined to the small intestine (SI), Th17 cells were also found in the colon after colonization with SFB, albeit at lower numbers (Figure S1A).

Although SFB colonization results in generation of Th17 cells in the small intestine, infections with intestinal pathogens such as *Citrobacter rodentium* that target the colon also induce Th17 responses (Mangan et al., 2006). To understand whether homeostatic SFB-elicited Th17 cells and pro-inflammatory *C.-rodentium*-elicited Th17 cells are induced with similar kinetics, we infected SFB-free *Il17a*^{Cre}*R26R*^{eYFP} mice with *C. rodentium* and followed Th17 cell induction in the colon on weeks 1, 2, and 4. Similarly to what was observed for SFB, the bacterial load of *C. rodentium* reached its peak in the first week and then progressively declined until it was cleared by week 4 (Figure 1C). Th17 cells induced by *C. rodentium* peaked at week 2, similarly to those induced by SFB, however *C.*-rodentium-elicited Th17 cells had not contracted by week 4 despite complete bacterial clearance (Figure 1D).

Th17 cells elicited by *C. rodentium* are known to produce large amounts of interferon-γ (IFN-γ) (Ahlfors et al., 2014). In contrast, Th17 cells generated upon SFB colonization produce mostly IL-17A (Ivanov et al., 2009), although a different study reported a modest increase in IFN-γ upon SFB colonization (Gaboriau-Routhiau et al., 2009). Due to differences in animal models, chosen time points and intestinal microbiota composition in these studies, it is difficult to draw conclusions regarding the cytokine profiles of Th17 cells elicited in these two conditions. For this reason, we compared cytokine production after *ex vivo* stimulation with phorbol myristate acetate (PMA) and ionomycin of eYFP^{+} Th17 cells from *Il17a*^{Cre}*R26R*^{eYFP} mice, colonized with either SFB or *C. rodentium*. Th17 cells generated in the first week mostly expressed IL-22 with IL-17A, or just IL-17A in either condition (Figures 2A and 2B) and started expressing IFN-γ, especially in *C.-rodentium*-infected mice, in the later phase of the infection (at 2 and 4 weeks), consistent with published reports on SFB- and *C.-rodentium*-elicited

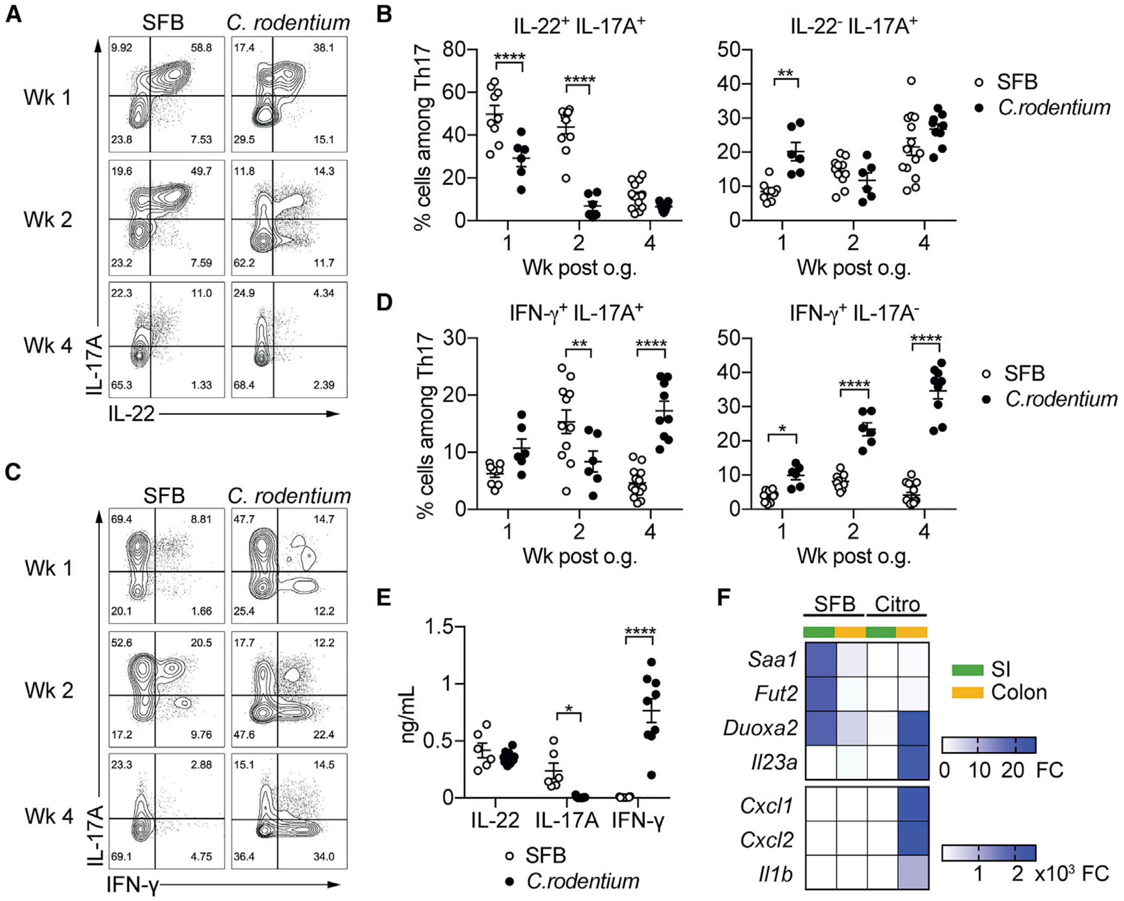

Figure 2. SFB- and *C.-rodentium*-Elicited Th17 Cells Have Different Cytokine Profiles

(A–D) Representative intracellular staining for IL-17A and IL-22 (A) or IFN-γ (C) and corresponding quantifications in (B) and (D) in Th17 cells from the SI of mice colonized with SFB and colon of mice infected with *C. rodentium* at 1 (n = 9 and 6), 2 (n = 11 and 6), and 4 (n = 14 and 9) weeks after gavage. Lamina propria cells were isolated at the indicated time point, re-stimulated with PMA and ionomycin and Brefeldin A for 2 h, and analyzed by FACS. Bars show the mean ± SEM for the indicated populations, and each symbol represents an individual mouse from two pooled independent experiments.

(E) Cytokine concentration in supernatants of FACS-purified Th17 cells from the SI of mice colonized with SFB or colon of mice infected with *C. rodentium*. In the graphs, bars show the mean ± SEM, and each symbol represents a technical replicate. Cells were FACS-purified from one sample per group, obtained by pooling 3–6 mice. Results are representative of three independent experiments.

(F) Fold-change (FC) induction of listed genes quantified by qPCR in the SI (green) and colon (orange) of mice colonized with SFB or infected with *C. rodentium*, 1 week after gavage. Fold change is calculated on the matching organs from untreated mice and gene expression is normalized against β-2-microglobulin. Data are from two pooled independent experiments. $^*p < 0.05$, $^{**}p < 0.01$, $^{****}p < 0.0001$ by two-way ANOVA with Sidak's post-test. Please see also Figure S2.

T cells (Atarashi et al., 2015). Although a transient induction of IFN-γ^+IL-17A$^+$ Th17 cells was observed in SFB colonized mice at 2 weeks after infection, the proportion of IFN-γ^+IL-17A$^-$ Th17 cells was significantly higher in *C. rodentium* colonized mice than in SFB colonized mice at all time points. (Figures 2C and 2D). SFB-elicited Th17 cells resident in the colon displayed a similar profile throughout the chosen time points compared with time points of homeostatic small intestinal Th17 cells (Figure S2A). In contrast *C.-rodentium*-elicited Th17 cells found in the small intestine diverged from their counterpart in the colon after the first week and showed a muted cytokine expression (Figure S2B). Although re-stimulation with PMA and ionomycin is a useful tool to identify subpopulations of Th17 cells based on their cytokine production, it does not necessarily reflect the actual production of the protein *in vivo*. For this reason, we FACS-sorted intestinal Th17 cells from *Il17a*^{Cre}*R26R*^{eYFP} mice colonized with SFB or *C. rodentium* 2 weeks after infection and cultured them without further stimulation to assess their production of cytokines on the protein level. Interestingly, IL-22 was secreted to a similar extent by SFB- and *C.-rodentium*-elicited Th17 cells, whereas IL17-A secretion was higher in SFB-induced Th17 cells, and increased amounts of IFN-γ were secreted only by *C.-rodentium*-elicited Th17 cells (Figure 2E).

In order to investigate what factors might mediate the differences in functionality between SFB- and *C.-rodentium*-elicited Th17 cells, we next determined whether SFB versus *C. rodentium* infection induces different molecules in intestinal tissue samples comprising epithelial and hematopoietic cells (Figures 2F and S2C).

As previously reported, SFB infection led to upregulation of serum amyloid A1 (*Saa1*) in the small intestinal tissue, which is

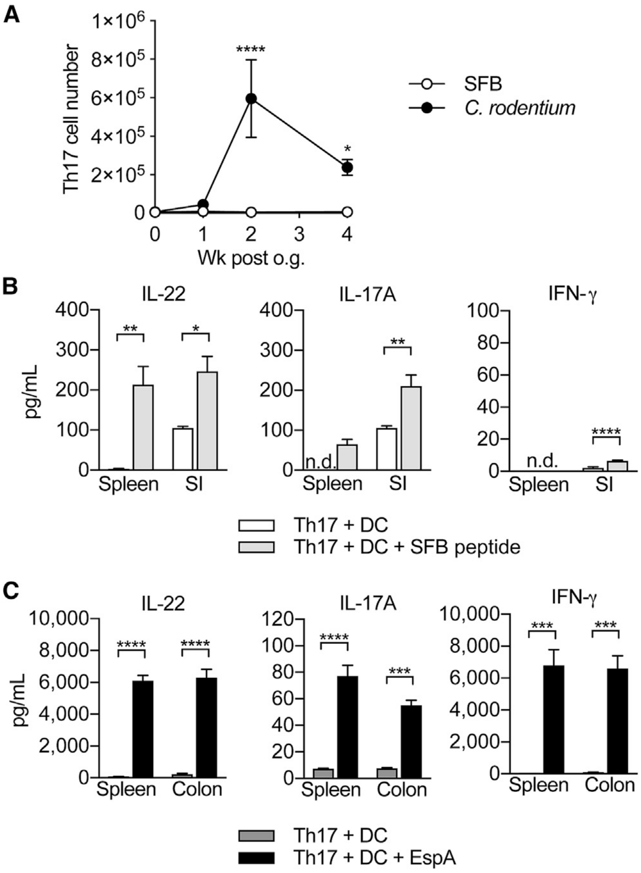

Figure 3. Peripheral Th17 Cells Retain Their Antigen-Specificity in Response to SFB and *C. rodentium*

Shown in (A) are the absolute numbers of Th17 cells in the spleen of mice colonized with SFB or infected with *C. rodentium* at 0 (n = 11 and 5), 1 (n = 10 and 6), 2 (n = 12 and 6), and 4 (n = 6 and 8) weeks after gavage. Symbols show the mean ± SEM. Cytokine concentration in supernatants of FACS-purified Th17 cells from the spleen and SI of mice colonized with SFB (B) or from the spleen and colon of mice infected with *C. rodentium* (C), cultured with DCs and SFB peptide (B) or EspA (C). Bars show the mean + SEM of technical replicates (n = 3–4). Cells were FACS-purified from one sample per group, obtained by pooling 3–6 mice. Results are representative of three (B) and two (C) independent experiments. *$p < 0.05$, **$p < 0.01$, ***$p < 0.001$, ****$p < 0.0001$ by two-way (A) or one-way (B and C) ANOVA with Sidak's (A) or Tukey's (B and C) post-test. n.d., not detected. See also Figure S3.

linked with the induction of Th17 cells (Sano et al., 2015). This was also observed, to a lesser extent, in the colon of SFB-colonized mice, but not in tissue samples from *C.-rodentium*-infected mice. Also characteristic for SFB was the induction of fructosyltransferase 2 (*Fut2*) which was shown to foster bacterial species that metabolize fucosylated substrates and protect against infection with *Salmonella typhimurium* (Schnupf et al., 2017). Dual oxidase maturation factor 2 (*Duoxa2*), the maturation factor for reactive oxygen species (ROS)-generating enzyme dual oxidase 2 (Duox2) was upregulated both in tissue from SFB- as well as *C.-rodentium*-infected mice. This is consistent with the role of ROS in the induction of SFB- as well as *C.-rodentium*-elicited intestinal Th17 cells (Atarashi et al., 2015). *Il23a*, *Il1b*, *Cxcl1*, and *Cxcl2* on the other hand were barely induced in intestinal tissue samples by SFB colonization, whereas they were highly expressed in colonic but not small intestinal tissue from *C.-rodentium*-infected mice.

Thus, infection with a commensal bacterium compared with a pathogen has distinct consequences for the response of epithelial and hematopoietic cells in mucosal tissue.

Cytokine Responses of Th17 Cells in the Periphery Recapitulate Their Intestinal Counterparts

We next investigated the dissemination of Th17 cells elicited by SFB or *C. rodentium* into the periphery over a time point of 4 weeks after colonization. SFB-elicited Th17 cells were detectable in the spleen, albeit at very low numbers. In contrast, *C.-rodentium*-elicited Th17 cells seeded the periphery in substantial numbers with a peak 2 weeks after infection, but still showed high numbers 4 weeks later (Figure 3A). FACS-sorted Th17 cells from the spleen and intestine were then cultured with dendritic cells (DCs) and immunodominant epitopes for either SFB or *C. rodentium* (EspA) to determine cytokine production in response to recall. SFB-induced Th17 cells isolated from the small intestine produced some IL-22 and IL-17A, but not IFN-γ even without re-stimulation, whereas those isolated from the spleen did not produce cytokines unless re-stimulated, but then recapitulated the specificity and cytokine profile of small intestinal Th17 cells (Figure 3B). *C.-rodentium*-induced Th17 cells isolated from the colon as well as the spleen produced concentrations of IL-22 and IL-17A protein upon re-stimulation, but in contrast to SFB-specific Th17 cells, they produced particularly high amounts of IFN-γ (Figure 3C). Thus, SFB-specific Th17 cells have very limited expansion potential and are found in very small numbers only in the periphery where re-stimulation by SFB peptide illustrates their origin and specificity as well as their imprinted cytokine profile. *C.-rodentium*-induced Th17 cells on the other hand distribute to the periphery in large numbers and exhibit an inflammatory cytokine profile indicative of plasticity towards a Th1-like cytokine pattern with high protein concentrations of IFN-γ.

Homeostatic Tissue-Resident Th17 Cells Do Not Clear *C. rodentium* Infection

Small intestinal Th17 cells that arise in response to commensal microbes such as SFB might be expected to display a "non-inflammatory" cytokine profile. However, SFB and intestinal Th17 cells have been implicated in inflammatory disorders outside the intestine, including EAE (Lee et al., 2011; Wu et al., 2010). To understand whether the presence of intestinal Th17 cells influences the course of EAE in our model of SFB re-introduction, we immunized SFB^+ and SFB^- mice with oligodendrocyte glycoprotein peptide in complete Freund's adjuvant (MOG-CFA) to induce EAE. Although clear differences in SFB abundance (Figure S3A) and numbers of intestinal Th17 cells (Figure S3B) were observed between the two groups, there was no difference in disease onset and severity after immunization with MOG-CFA (Figure S3C). Furthermore, frequency of Th17 cells in the spinal cord (Figure S3D) did not vary between SFB^+ and SFB^- mice. This suggests that induction of inflammatory Th17 cell responses in the periphery are not influenced by resident intestinal Th17 cells and the presence of SFB.

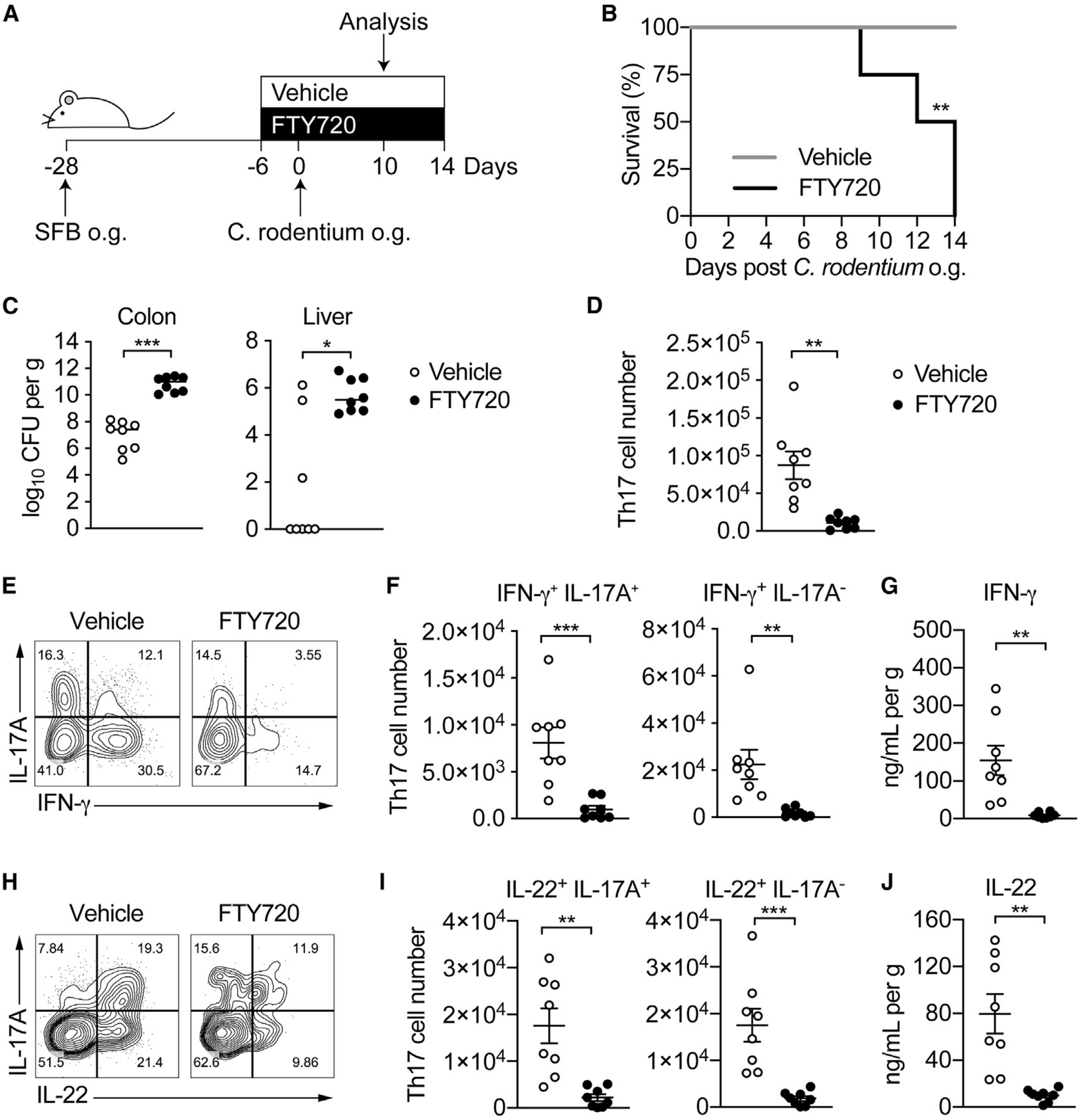

Figure 4. Homeostatic Tissue-Resident Intestinal Th17 Cells Do Not Clear *C. rodentium* Infection

(A) Schematic drawing of the experimental protocol. Mice were orally gavaged with SFB^+ feces 4 weeks before the infection with *C. rodentium* (at day 0). Six days before the infection with *C. rodentium* (day −6), mice were intra-peritoneally injected with FTY720 (3 mg/kg) or control vehicle every day. FTY720 and control vehicle administration continued after the infection every other day until the end of the experiment.

(B) Survival curve of *C.-rodentium*-infected mice treated with vehicle or FTY720.

(C) *C. rodentium* burdens in colon and liver.

(D–J) Absolute numbers of colonic Th17 cells. Representative intracellular staining for IL-17A and IFN-γ (E) or IL-22 (H) and quantification (F and I) in colonic Th17 cells. IFN-γ (G) and IL-22 (J) protein content in colon explant cultures. Bars show median (C) or mean ± SEM (D, F, G, I, and J) and each symbol represents an individual mouse (n = 8). Results are representative of three independent experiments. *$p < 0.05$, **$p < 0.01$, ***$p < 0.001$ by Mantel-Cox (B), Mann-Whitney (C) or Student's t test (D, F, G, I, and J). See also Figure S4.

However, Th17 cells are clearly involved in inflammatory processes within the intestinal environment, such as IBD, but it remains to be determined whether pro-inflammatory Th17 cells contributing to intestinal inflammation are Th17 cells *de novo* generated under pro-inflammatory conditions or rather tissue-resident "homeostatic" Th17 cells elicited by SFB that acquire a pro-inflammatory phenotype. To address this question, we colonized mice with SFB to induce differentiation of tissue-resident homeostatic Th17 cells in the intestine and then infected them with *C. rodentium*. Intestinal Th17 cell differentiation is initiated in the mesenteric lymph node (mLN) and the cells subsequently migrate to the intestinal lamina propria (Schreiber et al., 2013). To block the migration of newly generated *C.*-rodentium-specific Th17 cells from the mLN to the intestine, we administered the drug FTY720 after SFB colonization (Murphy et al., 2012), as illustrated in Figure 4A. FTY720 is an

analogue of sphingosine-1-phosphate, and its administration prevents lymphocyte egress from the lymph nodes and trafficking to target tissues (Chiba et al., 2006).

FTY720 treatment did not affect the numbers or cytokine profile of SFB-elicited Th17 cells in the colon, and they remained at a baseline number of 3.7×10^3 cells (Figure S4A–S4C).

Upon infection with *C. rodentium,* SFB-colonized mice treated with FTY720 succumbed to the infection between day 9 and 14 (Figure 4B) and displayed higher bacterial loads in the colon and dissemination to the liver compared than mice receiving only the vehicle (Figure 4C). Vehicle-treated mice infected with *C. rodentium* showed an increase in the Th17 population to 8.7×10^4 cells. Administration of FTY720 resulted in a marked decrease in numbers of colonic Th17 cells (Figure 4D), back to baseline numbers seen after Th17 cell induction by SFB (Figure S1A). This suggested that SFB-elicited Th17 cells resident in the colon did not expand in response to *C. rodentium*. Moreover, a smaller number of colonic Th17 cells from FTY720-treated mice were IFN-γ^+IL-17A$^+$ and IFN-γ^+IL-17A$^-$ compared with Th17 cells from mice treated with the vehicle (Figures 4E and 4F).

Consistently, production of IFN-γ was markedly decreased in the colons of mice receiving the drug (Figure 4G). Thus, Th17 cells elicited by SFB and resident in the colon of FTY720-treated mice did not acquire the "classical" Th17 pro-inflammatory phenotype observed in Th17 cells elicited by *C. rodentium* infection, whereas the latter could not contribute IFN-γ because their migration to the colon was blocked by FTY720. Furthermore, the proportion of IL-22-producing cells and protein concentration of IL-22 were substantially reduced (Figure 4H–4J) in line with the finding that the major source of IL-22 beyond day 5 after infection are adaptive T cells with a Th17 cell origin (Ahlfors et al., 2014; Basu et al., 2012). However, the number of ILC3s, which supply IL-22 prior to the induction of the adaptive response, was not affected by FTY720 treatment in *C.-rodentium*-infected mice (Figure S4D).

As *C.-rodentium*-specific Th17 cells were blocked from entry into the intestine, the infection could not be contained, and the mice succumbed to bacterial dissemination.

Homeostatic Tissue-Resident and Inflammatory Intestinal Th17 Cells Are Characterized by Distinct Metabolism

To characterize the molecular state associated with the different functional activities of Th17 cells induced by commensal SFB versus the pathogen *C. rodentium*, we profiled the transcriptome of these two populations by using RNA-sequencing (RNA-seq). We colonized mice with either SFB or *C. rodentium* and harvested Th17 cells from both small intestine and colon after 2 weeks at the peak of Th17 cell expansion. Hierarchical clustering revealed that the greatest variations in gene expression occurred between Th17 cells isolated from the colon of *C.-rodentium*-infected mice and the remaining three groups, including Th17 cells from the small intestine and colon of SFB-colonized mice and from the small intestine of *C.-rodentium*-infected mice (Figure 5A). *C.-rodentium*-elicited Th17 cells from the SI displayed higher similarity to homeostatic Th17 from the SI and colon of mice colonized with SFB than to inflammatory Th17 cells from the colon of mice colonized with *C. rodentium*. (Figure 5A). In correspondence with what was previously observed at the protein level (Figure 2E), small intestinal SFB-elicited Th17 cells expressed higher read counts for *Il17a* and comparable read counts for *Il22* compared with pro-inflammatory *C.-rodentium*-derived colonic Th17 cells. Furthermore, reads for T-box-containing protein expressed in T cells (*Tbx21)* and *Ifng*, which are expressed by Th17 cells that switch towards a pro-inflammatory profile, were increased in *C.-rodentium*- but not SFB-induced colonic Th17 cells (Figure 5B).

The natural habitat for SFB is the small intestine, whereas *C. rodentium* colonizes the colon. Considering the high degree of similarity between SFB-elicited Th17 cells from the colon and the small intestine, we asked whether the comparison between these two homeostatic populations and inflammatory *C.-rodentium*-derived colonic Th17 cells would result in similar sets of differentially expressed genes. As shown in Figure 5C, the vast majority of differentially expressed genes appeared in both comparisons: SFB-derived colonic Th17 cells versus *C.-rodentium*-derived colonic Th17 cells and SFB-derived small intestinal Th17 cells versus *C.-rodentium*-derived colonic Th17 cells. Moreover, this similarity trend persisted also in the non-differentially expressed genes with only a handful of genes exhibiting opposite trends in the two comparisons. For this reason, we decided to use SFB-elicited colonic Th17 cells as our homeostatic control population and compared it to inflammatory *C.-rodentium*-elicited colonic Th17 cells.

A consistent observation for SFB-elicited Th17 cells was the inhibition of pro-inflammatory pathways, including Janus kinase (JAK) and signal transducers and activators of transcription (STAT) signaling, nitric oxide, and ROS production compared with that in *C.-rodentium*-derived Th17 cells (Figure 5D). In contrast, anti-inflammatory pathways, such as sumoylation, phosphatase and tensin homolog (PTEN), and sirtuin signaling were activated in SFB-derived Th17 cells. Expression of *Sirt1*, which belongs to the sirtuin family of NAD$^+$-dependent deacetylases, is downregulated in IBD biopsies and increased in mucosal samples of IBD patients successfully treated with Infliximab (Caruso et al., 2014).

Transcriptional regulators and chromatin modifiers involved in regulating cell growth, differentiation, and apoptosis such as *Rhob, Znf217*, *Rnd3*, *Xiap*, *EP300*, and *Sp1* were upregulated in SFB-induced Th17 cells (Figure S5A) as were signaling modifiers, cell cycle inhibitors, and anti-apoptotic factors connected to the PTEN pathway (Figure S5B). A further indication of the less inflammatory nature of SFB-elicited Th17 cells is the enrichment for mitochondria-encoded genes involved in oxidative phosphorylation (OXPHOS) (*mt-Nd1*, *mt-Nd2*, *mt-Nd3*, *mt-Nd4*, *mt-Nd4l*, and *mt-Nd6*) compared with markers of gluconeogenesis and glycolysis such as *Crtc2* and *Ldha*, respectively, that are downregulated in SFB-elicited Th17 cells but highly expressed on inflammatory *C.-rodentium*-elicited Th17 cells (Figure S5C).

In order to investigate whether our RNA-seq data from *ex vivo* intestinal Th17 cells show any overlap with *in vitro* generated "pathogenic" versus "non-pathogenic" Th17 cells, we compared our dataset from colonic SFB- versus *C.-rodentium*-elicited Th17 cells to published gene expression data from Th17 cells differentiated in the presence of transforming growth factor-β (non-pathogenic Th17) or IL-23 (pathogenic Th17) (Ghoreschi et al., 2010). Out of 197 genes differentially expressed between

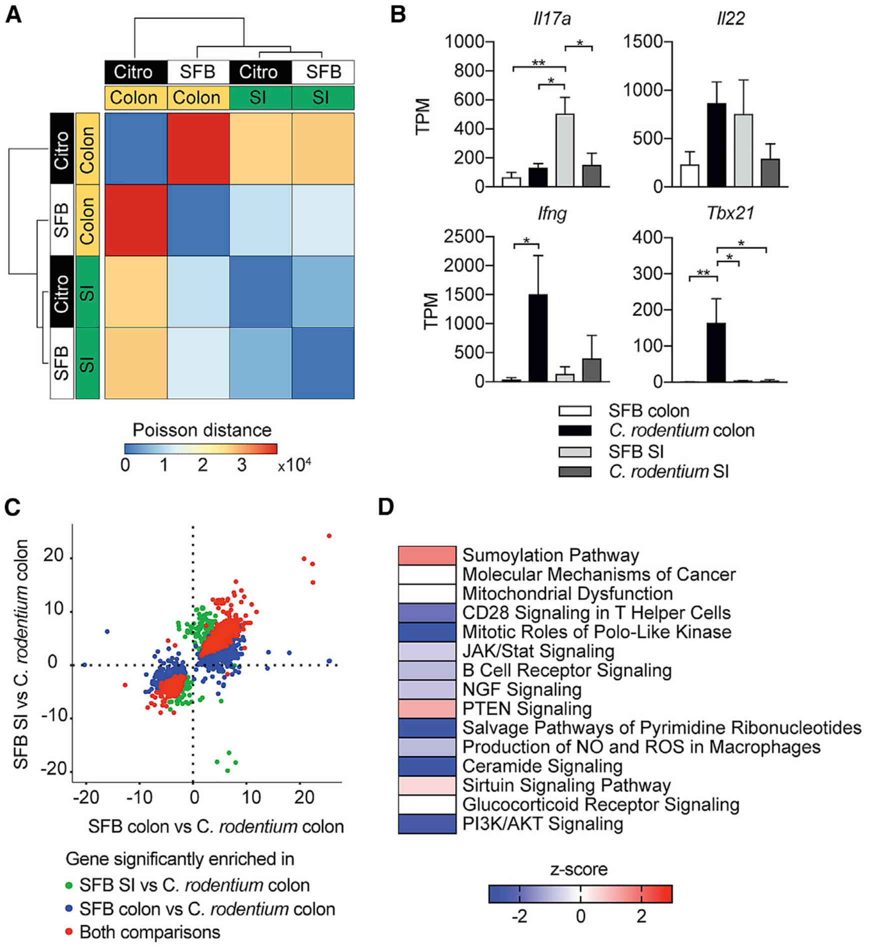

Figure 5. Intestinal Th17 Cells Elicited by SFB and *C. rodentium* Have Distinct Transcriptional Profiles

(A) RNA-sequencing analysis of Th17 cells from the SI and colon of *C. rodentium* (Citro)-infected and SFB-colonized mice, presented as heatmap of condition similarity showing Poisson distance between experimental groups.

(B) Gene expression in Th17 cells from the SI and colon of *C.-rodentium*-infected and SFB-colonized mice, presented as normalized read counts (TPM).

(C) Scatter plot of differentially expressed genes (log_2-fold change) in SFB SI versus *C. rodentium* colon against SFB colon versus *C. rodentium* colon. Each dot represents a gene differentially expressed (adjusted $p < 0.05$) in at least one comparison. Specifically, genes in green are significantly different in the SFB SI versus *C. rodentium* colon comparison; genes in blue are significantly different in SFB colon versus *C. rodentium* colon comparison; and genes in red are significantly different in both comparisons.

(D) Top 15 canonical pathways in colonic SFB- versus *C. rodentium*-induced Th17 cells identified by ingenuity pathway analysis (IPA) and ranked on p value. The colors indicate activation *Z*-score of each pathway with red corresponding to positive *Z*-scores, blue to negative *Z*-scores, and white to no activity pathway available. *$p < 0.05$, **$p < 0.01$ by one-way ANOVA with Tukey's post-test. See also Figure S5.

pathogenic and non-pathogenic *in-vitro*-differentiated Th17 cells, 189 genes were detectable in colonic SFB- or *C.-rodentium*-elicited Th17 cells. Among these 189 genes (depicted in Figure S5D), 33 genes were differentially expressed to a significant extent in our dataset and followed a consistent trend in tissue-resident, SFB-elicited Th17 cells compared with *in vitro* "regulatory" Th17 cells as well as in *C.-rodentium*-elicited Th17 cells compared with "pathogenic" Th17 cells (Figures S5D–S5F). Markers previously observed in "non-pathogenic" versus "pathogenic" *in-vitro*-differentiated Th17 cells, such as *Maf*, *Il10*, or *Tbx21* were differentially expressed in SFB versus *C. rodentium* Th17 cells, respectively (Figure S5D–S5F).

Further focus on the metabolism in the two Th17 cell types illustrated that *C.-rodentium*-elicited Th17 cells were enriched in metabolic pathways such as pyruvate metabolism, amino acid biosynthesis, purine, and pyrimidine metabolisms (Figure 6A). The usage of these metabolic pathways is consistent with the behavior of activated T cells, which display increased capacity for glucose uptake, pyruvate generation, and demand for nucleotides and other biomass to support their rapid proliferation and generation of effector molecules (Olenchock et al., 2017). Effector T cells also increase mitochondrial OXPHOS upon activation, together with anaerobic glycolysis, although the latter remain the most substantially increased metabolism in these cells (van der Windt et al., 2012; Wang et al., 2011). In accordance with this observation, expression profile analysis indicated an upregulation of both OXPHOS and glycolytic pathways in *C.-rodentium*-induced Th17 cells, whereas tissue-resident homeostatic Th17 cells seemed to rely mainly on OXPHOS (Figure 6A). To further investigate metabolic phenotypes of tissue-resident homeostatic and *C.-rodentium*-elicited Th17 cells, we used reporter metabolite analysis and identified those metabolites around which transcriptional changes occur (see STAR Methods). This analysis showed that tissue-resident homeostatic Th17 cells upregulated reducing agents, including nicotinamide adenine dinucleotide phosphate (NADPH) and glutathione (GSH) (Figure 6B and Table S1), which are crucial to balance the oxidative stress generated by OXPHOS. *C.-rodentium*-elicited Th17 cells, on the other hand, upregulated energy metabolites in the cytosol (Figure 6B and Table S1) and genes encoding glycolytic enzymes (*Hk1*, *Pfkl*, *Aldoa*, *Eno1*, *Pkm*, and *Ldha*) (Figure 6C).

Given that the RNA-seq data suggested pronounced metabolic differences between the tissue-resident and infection-induced Th17 cells, we carried out extracellular flux analysis in order to define the bioenergetic profiles of these populations. The basal oxygen consumption rate (OCR), an indicator of OXPHOS, was slightly higher in *C.-rodentium*- than in SFB-derived intestinal Th17 cells (Figure 7A). Furthermore, we observed a significant increase of extracellular acidification rate (ECAR), an indicator of glycolysis, in *C.-rodentium*-elicited

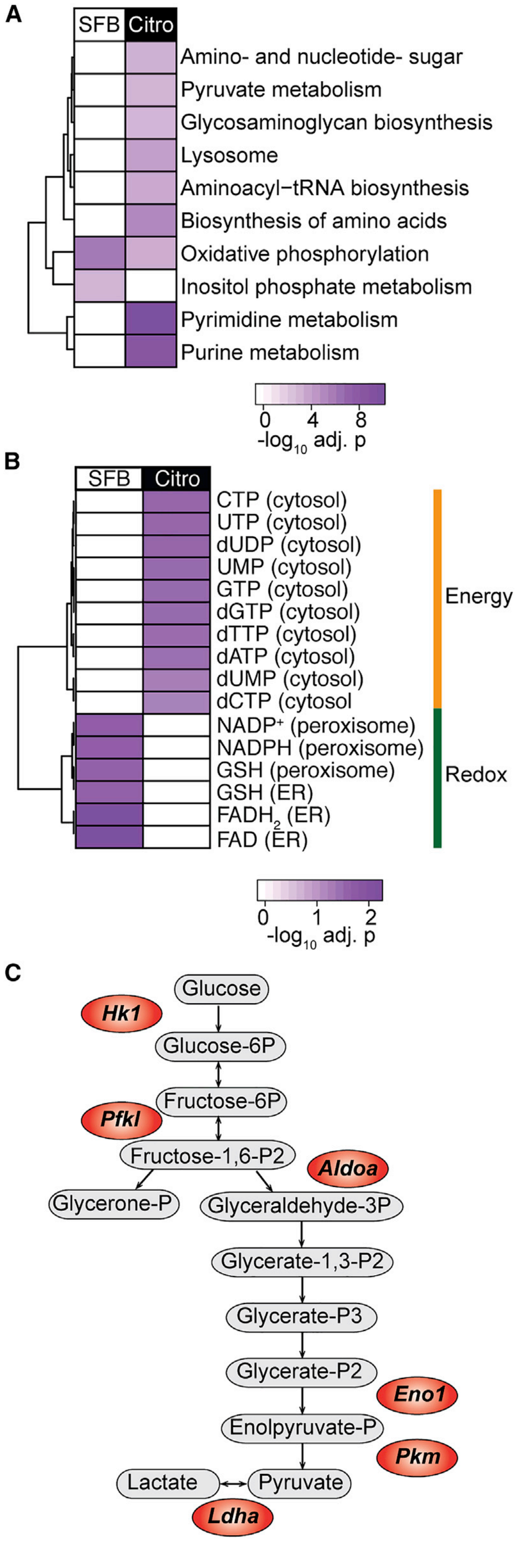

Figure 6. SFB- and *C.-rodentium*-elicited intestinal Th17 cells display different metabolic phenotypes

(A) Kyoto Encyclopedia of Genes and Genomes (KEGG) metabolic pathways enriched in SFB- and *C.-rodentium*-elicited colonic Th17 cells, on the basis of upregulated genes with adjusted $p < 0.001$.

(B) Selection of differentially upregulated reporter metabolites involved in energy generation and redox reactions in SFB- and *C.-rodentium*-elicited colonic Th17 cells (adjusted $p < 0.05$).

(C) Metabolism-related genes increased in *C.-rodentium*-induced colonic Th17 cells (adjusted $p < 0.05$) (in red) as well as the associated reactions involved in glycolysis. See also Table S1.

Th17 cells (Figure 7B). These cells also displayed higher ATP-coupled respiration than did SFB-derived Th17 cells (Figure 7C), in agreement with the reporter metabolite analysis in Figure 6B. Collectively, these data support the findings from our RNA-seq analysis and suggest that *C.-rodentium*-derived Th17 cells exhibit an energetic glycolytic phenotype, whereas SFB-derived Th17 cells show a resting aerobic profile, mainly dependent on OXPHOS (Figure 7D).

Previous reports have linked changes in mitochondrial morphology to different metabolic phenotypes (Rambold and Pearce, 2018). Analysis of the mitochondrial network by confocal microscopy showed that SFB-elicited Th17 cells displayed an elongated mitochondrial morphology (Figure 7E). In contrast, in *C.-rodentium*-elicited Th17 cells, there was a significantly higher fraction of individual (not linked to a network) and shorter mitochondria than in SFB-elicited cells (Figures 7E–7G). Nonetheless, we did not detect changes in mitochondrial content (evaluated as total mitochondrial area per cell) (Figure 7H). These results are in line with previous findings associating a tubular and connected mitochondrial network with cells relying mainly in OXPHOS and a more fragmented morphology with cellular populations exhibiting higher glycolytic activity (Buck et al., 2016; Mishra and Chan, 2016).

Altogether, these data confirmed that tissue-resident, SFB-elicited Th17 cells, in contrast to *C. rodentium*-induced Th17 cells, showed muted effector functions and a metabolism that supports the attenuated energy requirements of this homeostatic population.

DISCUSSION

Th17 cells are considered important players in the orchestration of inflammatory and autoimmune diseases. Multiple therapeutic approaches targeting Th17 cells or their cytokine IL-17 have shown promising results in ameliorating inflammation (Patel and Kuchroo, 2015; Sherlock et al., 2012). However, what is rarely taken into consideration is the fact that gut-indigenous Th17 cells are important for barrier protection and might be victims of therapeutic approaches with potential deleterious long-term effects. An example is the unsuccessful targeting of IL-17 in Crohn's patients, which resulted in increased adverse effects (Hueber et al., 2012). However, it is clear that Th17 cells are also linked to intestinal inflammatory diseases such as IBD, where they are highly enriched and linked to several genetic risk loci (Kempski et al., 2017; Knights et al., 2013).

In this study, we have set out to define the differences in intestinal Th17 cells that are either tissue-resident and beneficial for barrier maintenance or induced by infection, effective at dealing with the threat but displaying a highly inflammatory profile.

In mice, Th17 cells in the gut are largely induced by commensal SFB (Ivanov et al., 2009), which play an important

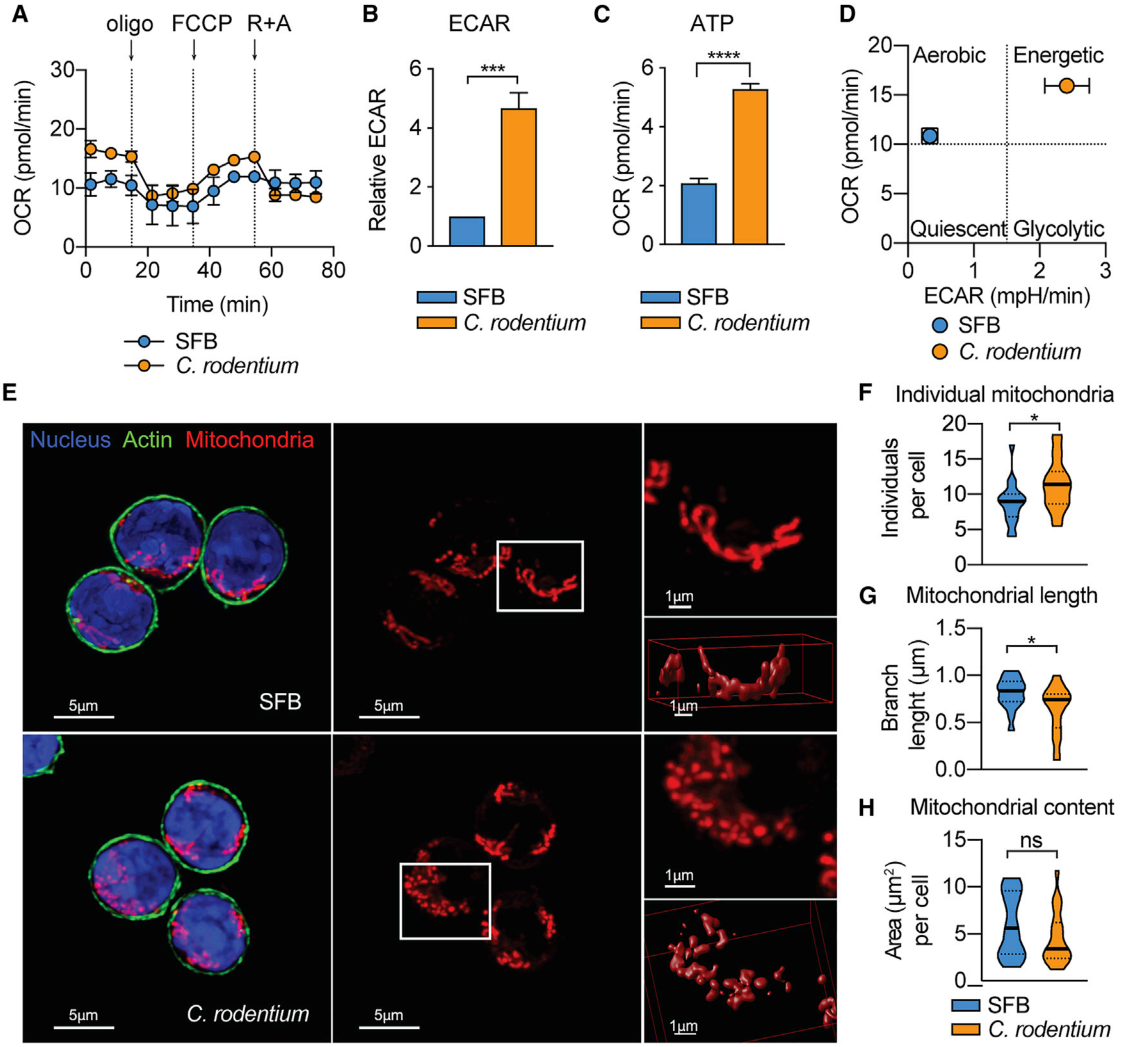

Figure 7. SFB- and *C.-rodentium*-Intestinal Th17 Cells Have Distinct Bioenergetic Profiles and Mitochondrial Morphology

Th17 cells from the SI of SFB-colonized mice and the colon of *C.-rodentium*-infected mice were FACS-sorted and used for further analysis.

(A) Oxygen consumption rate (OCR) at baseline and in response to oligomycin (Oligo), carbonyl cyanide 4-(trifluoromethoxy) phenylhydrazone (FCCP), and rotenone plus antimycin (R + A).

(B) Baseline extracellular acidification rate (ECAR) in relation to ECAR of SFB-elicited Th17 cells.

(C) Measurement of ATP-coupled respiration.

(D) OCR (measure of OXPHOS) plotted against ECAR (measure of glycolysis). Data are from 2–3 pooled independent experiments and are shown as mean ± SEM ***$p < 0.001$, ****$p < 0.0001$ by Student's t test.

(E–H) *Z*-stack images were used to evaluate mitochondrial morphology by confocal microscopy. Mitochondria were stained with MitoTracker Red CMXRos (red), nuclei with DAPI (blue), and actin with Alexa Fluor 488 phalloidin (green). A magnification (top) and a 3D surface rendered image (bottom) of the area indicated in white are shown (E). Quantification of individual mitochondria (F), mean mitochondrial branch length (G), and mitochondrial area per cell (H) obtained using MiNa Macro and Fiji software. In the violin plot, bars show the median and dotted bars the quartiles (F–H). Data are representative of two independent experiments with at least 50 cells analyzed per condition. *$p < 0.05$ by Student's t test. n.s., not significant. Scale bars, 1 μm and 5 μm.

role in gut homeostasis, the development of the mucosal architecture (Umesaki et al., 1995), as well as induction of immunoglobulin A (IgA) and post-natal maturation of gut immune functions, but are apathogenic (Gaboriau-Routhiau et al., 2009; Klaasen et al., 1993; Schnupf et al., 2013; Schnupf et al., 2017). Thus, the response to SFB that leads to the development of Th17 cells proceeds without epithelial cell damage and inflammatory infiltration of the lamina propria (Caballero and Pamer, 2015). Using our IL-17A fate reporter mice (Hirota et al., 2011), we noticed that after the extensive re-derivation program of mice prior to the start of the Francis Crick Institute, we had lost SFB and could not detect Th17 cells in the small intestine of our reporter mice. Upon introduction of fecal material from germ-free mice monocolonized with SFB (provided by Cerf-Bensussan), Th17 cells re-colonized the intestine of mice, reaching their maximal induction 2 weeks later. We therefore took advantage of this situation to follow and characterize the development of intestinal Th17 cells induced

by SFB. Like SFB, the intestinal pathogen *C. rodentium* tightly adheres to epithelium, but unlike SFB causes epithelial cell death through effectors injected via a type III secretion system (Crepin et al., 2016), resulting in the induction of an inflammatory Th17 cells response (Torchinsky et al., 2009). Inflammatory Th17 cells are highly plastic and tend to expand their cytokine repertoire to include IFN-γ, even losing expression of IL-17A as we could determine with our fate reporter system.

Our data show that SFB-induced Th17 cells do not have extensive plasticity and rarely switch on IFN-γ in contrast to Th17 cells that were induced after infection with *C. rodentium*. In fact, a previous study found that resident intestinal Th17 cells transdifferentiate into a regulatory phenotype under inflammatory conditions rather than contribute to inflammation (Gagliani et al., 2015). Under steady-state conditions, however, we did not observe any acquisition of regulatory T cell markers.

A potential caveat for our studies was the tissue-specific localization of these two bacteria, with SFB resident in the small intestine, whereas *C. rodentium* colonizes the colon. Nevertheless, SFB-elicited Th17 cells are also found in the colon, albeit in smaller numbers than in the small intestine, and Th17 cells induced by *C. rodentium* infection can be found in the small intestine. In our RNA-seq comparison of Th17 cells induced by these different bacteria, we therefore carefully compared both colonic and small intestinal Th17 cells for both conditions. Indeed, SFB-elicited Th17 cells in the colon were very similar to those resident in the small intestine. Th17 cells elicited by *C. rodentium* infection but resident in the small intestine had little in common with the inflammatory Th17 cell population in the colon and rather resembled the muted small intestinal population that arises after SFB colonization, whereas the colonic Th17 population induced after infection was different from all other conditions. Given that we also observed very low mRNA expression of *Il23a* in the small intestine compared with that in the colon, this might explain the reduced cytokine expression of *C.-rodentium*-elicited Th17 cells, whose maintenance is dependent on this cytokine.

We therefore focused our analysis on comparing colon-resident Th17 cells either elicited by SFB or by *C. rodentium*.

A series of studies have implicated SFB and the associated Th17 cell response in systemic inflammatory conditions such as EAE or arthritis (Lee et al., 2011; Wu et al., 2010). A caveat for these studies is that they used germ-free mice, which are generally deficient in mounting effective immune responses as baseline. Our comparison of mice with diverse microbiota and that contained SFB or no SFB did not indicate any difference in the development of EAE whether or not SFB and intestinal Th17 cells were present. This is in line not only with the specificity of intestinal Th17 cells for SFB (Yang et al., 2014), but also with the fact that induction of EAE is dependent on *de-novo*-induced Th17 cells in regional lymph nodes after the immunization protocol. Likewise, our data, using inhibition of egress from draining lymph nodes via administration of FTY720, support the assumption that inflammatory Th17 cells induced by infection with *C. rodentium* arose *de novo* from naive CD4 T cells that then migrated to the colon from local lymph nodes rather than from the expansion of pre-existing resident SFB-elicited Th17 cells.

Although it would be difficult to distinguish resident barrier protective Th17 cells from infection-induced Th17 cells in standard wild-type mice that are bred in SFB-containing conditions, there are distinctions between the two states of Th17 cells that might inform therapeutic targeting of inflammatory Th17 cells. For instance, our group and others reported that intestinal Th17 cells, unlike inflammatory Th17 cells, are not dependent on IL-23 (Cua et al., 2003; Hirota et al., 2013) so that therapies targeting IL-23 would not endanger indigenous small intestinal Th17 cells. Consistently, mutations in the IL-23 receptor are associated with increased IBD risk and increased protein amounts of IL-23 are observed in subjects with IBD (Jostins et al., 2012; Knights et al., 2013).

Our RNA-seq analysis highlighted profound differences in metabolic conditions for tissue-resident versus inflammatory Th17 cells, showing that tissue-resident homeostatic Th17 cells mainly use OXPHOS, similarly to quiescent or memory T cells. This metabolic state was also reflected in the morphology of their mitochondria that resembled those previously reported in resting memory T cells. Previous data suggested that Th17 cells, like other T cell effector subsets and unlike regulatory T cells, are highly glycolytic. However, these data were generated exclusively from *in-vitro*-differentiated Th17 cells (Michalek et al., 2011).

It is likely that, in order to support rapid proliferation and effector functions, infection-induced Th17 cells predominantly use glycolysis, as shown in the RNA-seq and metabolic flux analyses. Therefore, targeting highly glycolytic Th17 cells is likely to spare small intestinal Th17 cells.

A number of studies have focused on defining characteristics of "pathogenic" Th17 cells, which were either derived from *in vitro* differentiation cultures or isolated from the CNS of mice with EAE (Gaublomme et al., 2015; Lee et al., 2012; Wang et al., 2015). Our analysis of intestinal Th17 cells did not corroborate previously identified markers thought to restrain (CD5L, also known as AIM) or promote (Gpr65, Plzp, and Toso) Th17 cell pathology. This might be due to the fact that *in-vitro*-differentiated Th17 cells do not adequately reflect the transcriptomic state of resident intestinal Th17 cells and, on the other hand, that Th17 cells involved in CNS inflammation are dissimilar to Th17 cells induced by an intestinal infection. A comparison of our dataset with previously published gene expression data (Ghoreschi et al., 2010) indicated minor overlap, but suggested that a small number of differentially expressed genes reported in non-pathogenic Th17 cells generated *in vitro* were found in those of tissue-resident Th17 cells, whilst some genes expressed in pathogenic Th17 cells were similarly expressed in infection-induced Th17 cells in our dataset.

In conclusion, it appears that resident intestinal Th17 cells, which in mice are almost exclusively induced by recognition of SFB, remain in a non-inflammatory, barrier protective state, display a muted metabolism and do not participate in systemic inflammatory responses.

Even though a small proportion of SFB-specific Th17 cells can be found in peripheral lymphoid organs, they display a resting memory phenotype. Th17 cells induced by a pathogen such as *C. rodentium*, however, generate a vigorous inflammatory response, display substantial plasticity and primarily engage aerobic glycolysis.

STAR★METHODS

Detailed methods are provided in the online version of this paper and include the following:

- KEY RESOURCES TABLE
- LEAD CONTACT AND MATERIALS AVAILABILITY
- EXPERIMENTAL MODEL AND SUBJECT DETAILS
  - Mice
- METHOD DETAILS
  - Colonization with Segmented Filamentous Bacteria
  - Quantitative PCR for Segmented Filamentous Bacteria
  - Quantitative PCR for intestinal tissue
  - Infection with Citrobacter rodentium
  - Cell isolation
  - Flow cytometry and cell sorting
  - Th17 cell culture
  - Recombinant proteins
  - EAE induction
  - Colon explant cultures
  - RNA-sequencing
  - Metabolic assays
  - Confocal microscopy
- QUANTIFICATION AND STATISTICAL ANALYSIS
- DATA AND CODE AVAILABILITY

SUPPLEMENTAL INFORMATION

Supplemental Information can be found online at https://doi.org/10.1016/j.immuni.2019.05.004.

ACKNOWLEDGEMENTS

This work was supported by the Francis Crick Institute, which receives its core funding from Cancer Research UK FC001092 to M.G.G. and FC001159 to B.S., The UK Medical Research Council FC001092 to M.G.G., FC001159 to B.S., and the Wellcome Trust FC001092 to M.G.G. and FC001159 to B.S. S.S. and S.L. are supported by the Engineering and Physical Sciences Research Council (EPSRC) EP/S001301/1.

We would like to acknowledge the Biological Research Facility at the Francis Crick Institute for expert breeding and maintenance of our mouse strains, the Flow Cytometry Facility and the Crick Advance Light Microscopy Facility. We are grateful to Drs. Nadine Cerf-Bensussan and Valerie Gaboriau-Routhiau (Inserm Paris) for fecal samples of germ-free mice monocolonized with SFB, and we thank Dr. Gordon Dougan (Sanger Institute Cambridge) for His-EspA-expressing *E. coli.*

AUTHOR CONTRIBUTIONS

S.O. designed, performed, and analyzed most of the experiments with input from A.M., A.I., M.T., and Y.L. M.G.G. provided advice on the metabolism experiments, and C.B. performed the metabolic measurements and mitochondrial analysis. S.L. and S.S. carried out computational analysis of metabolism from the RNA-seq data. G.K. and P.C. carried out bioinformatics analysis. B.S. conceived the project and wrote the manuscript.

DECLARATION OF INTERESTS

The authors declare no competing interests.

Received: October 2, 2018
Revised: February 28, 2019
Accepted: May 14, 2019
Published: June 19, 2019

REFERENCES

Ahlfors, H., Morrison, P.J., Duarte, J.H., Li, Y., Biro, J., Tolaini, M., Di Meglio, P., Potocnik, A.J., and Stockinger, B. (2014). IL-22 fate reporter reveals origin and control of IL-22 production in homeostasis and infection. J. Immunol. *193*, 4602–4613.

Atarashi, K., Tanoue, T., Ando, M., Kamada, N., Nagano, Y., Narushima, S., Suda, W., Imaoka, A., Setoyama, H., Nagamori, T., et al. (2015). Th17 cell induction by adhesion of microbes to intestinal epithelial cells. Cell *163*, 367–380.

Basu, R., O'Quinn, D.B., Silberger, D.J., Schoeb, T.R., Fouser, L., Ouyang, W., Hatton, R.D., and Weaver, C.T. (2012). Th22 cells are an important source of IL-22 for host protection against enteropathogenic bacteria. Immunity *37*, 1061–1075.

Buck, M.D., O'Sullivan, D., Klein Geltink, R.I., Curtis, J.D., Chang, C.H., Sanin, D.E., Qiu, J., Kretz, O., Braas, D., van der Windt, G.J., et al. (2016). Mitochondrial dynamics controls T cell fate through metabolic programming. Cell *166*, 63–76.

Caballero, S., and Pamer, E.G. (2015). Microbiota-mediated inflammation and antimicrobial defense in the intestine. Annu. Rev. Immunol. *33*, 227–256.

Caruso, R., Marafini, I., Franzè, E., Stolfi, C., Zorzi, F., Monteleone, I., Caprioli, F., Colantoni, A., Sarra, M., Sedda, S., et al. (2014). Defective expression of SIRT1 contributes to sustain inflammatory pathways in the gut. Mucosal Immunol. *7*, 1467–1479.

Chiba, K., Matsuyuki, H., Maeda, Y., and Sugahara, K. (2006). Role of sphingosine 1-phosphate receptor type 1 in lymphocyte egress from secondary lymphoid tissues and thymus. Cell. Mol. Immunol. *3*, 11–19.

Chung, H., Pamp, S.J., Hill, J.A., Surana, N.K., Edelman, S.M., Troy, E.B., Reading, N.C., Villablanca, E.J., Wang, S., Mora, J.R., et al. (2012). Gut immune maturation depends on colonization with a host-specific microbiota. Cell *149*, 1578–1593.

Crepin, V.F., Collins, J.W., Habibzay, M., and Frankel, G. (2016). Citrobacter rodentium mouse model of bacterial infection. Nat. Protoc. *11*, 1851–1876.

Cua, D.J., Sherlock, J., Chen, Y., Murphy, C.A., Joyce, B., Seymour, B., Lucian, L., To, W., Kwan, S., Churakova, T., et al. (2003). Interleukin-23 rather than interleukin-12 is the critical cytokine for autoimmune inflammation of the brain. Nature *421*, 744–748.

Endo, Y., Asou, H.K., Matsugae, N., Hirahara, K., Shinoda, K., Tumes, D.J., Tokuyama, H., Yokote, K., and Nakayama, T. (2015). Obesity drives Th17 cell differentiation by inducing the lipid metabolic kinase, ACC1. Cell Rep. *12*, 1042–1055.

Gaboriau-Routhiau, V., Rakotobe, S., Lécuyer, E., Mulder, I., Lan, A., Bridonneau, C., Rochet, V., Pisi, A., De Paepe, M., Brandi, G., et al. (2009). The key role of segmented filamentous bacteria in the coordinated maturation of gut helper T cell responses. Immunity *31*, 677–689.

Gagliani, N., Amezcua Vesely, M.C., Iseppon, A., Brockmann, L., Xu, H., Palm, N.W., de Zoete, M.R., Licona-Limón, P., Paiva, R.S., Ching, T., et al. (2015). Th17 cells transdifferentiate into regulatory T cells during resolution of inflammation. Nature *523*, 221–225.

Gaublomme, J.T., Yosef, N., Lee, Y., Gertner, R.S., Yang, L.V., Wu, C., Pandolfi, P.P., Mak, T., Satija, R., Shalek, A.K., et al. (2015). Single-cell genomics unveils critical regulators of Th17 cell pathogenicity. Cell *163*, 1400–1412.

Ghoreschi, K., Laurence, A., Yang, X.P., Tato, C.M., McGeachy, M.J., Konkel, J.E., Ramos, H.L., Wei, L., Davidson, T.S., Bouladoux, N., et al. (2010). Generation of pathogenic T(H)17 cells in the absence of TGF-β signalling. Nature *467*, 967–971.

Hirota, K., Duarte, J.H., Veldhoen, M., Hornsby, E., Li, Y., Cua, D.J., Ahlfors, H., Wilhelm, C., Tolaini, M., Menzel, U., et al. (2011). Fate mapping of IL-17-producing T cells in inflammatory responses. Nat. Immunol. *12*, 255–263.

Hirota, K., Turner, J.E., Villa, M., Duarte, J.H., Demengeot, J., Steinmetz, O.M., and Stockinger, B. (2013). Plasticity of Th17 cells in Peyer's patches is responsible for the induction of T cell-dependent IgA responses. Nat. Immunol. *14*, 372–379.

Huang da, W., Sherman, B.T., and Lempicki, R.A. (2009). Systematic and integrative analysis of large gene lists using DAVID bioinformatics resources. Nat. Protoc. *4*, 44–57.

Hueber, W., Sands, B.E., Lewitzky, S., Vandemeulebroecke, M., Reinisch, W., Higgins, P.D., Wehkamp, J., Feagan, B.G., Yao, M.D., Karczewski, M., et al.; Secukinumab in Crohn's Disease Study Group (2012). Secukinumab, a human anti-IL-17A monoclonal antibody, for moderate to severe Crohn's disease: unexpected results of a randomised, double-blind placebo-controlled trial. Gut *61*, 1693–1700.

Ivanov, I.I., Atarashi, K., Manel, N., Brodie, E.L., Shima, T., Karaoz, U., Wei, D., Goldfarb, K.C., Santee, C.A., Lynch, S.V., et al. (2009). Induction of intestinal Th17 cells by segmented filamentous bacteria. Cell *139*, 485–498.

Jostins, L., Ripke, S., Weersma, R.K., Duerr, R.H., McGovern, D.P., Hui, K.Y., Lee, J.C., Schumm, L.P., Sharma, Y., Anderson, C.A., et al.; International IBD Genetics Consortium (IIBDGC) (2012). Host-microbe interactions have shaped the genetic architecture of inflammatory bowel disease. Nature *491*, 119–124.

Kempski, J., Brockmann, L., Gagliani, N., and Huber, S. (2017). T_H17 cell and epithelial cell crosstalk during inflammatory bowel disease and carcinogenesis. Front. Immunol. *8*, 1373.

Klaasen, H.L., Van der Heijden, P.J., Stok, W., Poelma, F.G., Koopman, J.P., Van den Brink, M.E., Bakker, M.H., Eling, W.M., and Beynen, A.C. (1993). Apathogenic, intestinal, segmented, filamentous bacteria stimulate the mucosal immune system of mice. Infect. Immun. *61*, 303–306.

Kleinewietfeld, M., Manzel, A., Titze, J., Kvakan, H., Yosef, N., Linker, R.A., Muller, D.N., and Hafler, D.A. (2013). Sodium chloride drives autoimmune disease by the induction of pathogenic TH17 cells. Nature *496*, 518–522.

Knights, D., Lassen, K.G., and Xavier, R.J. (2013). Advances in inflammatory bowel disease pathogenesis: linking host genetics and the microbiome. Gut *62*, 1505–1510.

Knutton, S., Rosenshine, I., Pallen, M.J., Nisan, I., Neves, B.C., Bain, C., Wolff, C., Dougan, G., and Frankel, G. (1998). A novel EspA-associated surface organelle of enteropathogenic Escherichia coli involved in protein translocation into epithelial cells. EMBO J. *17*, 2166–2176.

Kumar, P., Monin, L., Castillo, P., Elsegeiny, W., Horne, W., Eddens, T., Vikram, A., Good, M., Schoenborn, A.A., Bibby, K., et al. (2016). Intestinal interleukin-17 receptor signaling mediates reciprocal control of the gut microbiota and autoimmune inflammation. Immunity *44*, 659–671.

Langmead, B., and Salzberg, S.L. (2012). Fast gapped-read alignment with Bowtie 2. Nat. Methods *9*, 357–359.

Lécuyer, E., Rakotobe, S., Lengliné-Garnier, H., Lebreton, C., Picard, M., Juste, C., Fritzen, R., Eberl, G., McCoy, K.D., Macpherson, A.J., et al. (2014). Segmented filamentous bacterium uses secondary and tertiary lymphoid tissues to induce gut IgA and specific T helper 17 cell responses. Immunity *40*, 608–620.

Lee, Y.K., Menezes, J.S., Umesaki, Y., and Mazmanian, S.K. (2011). Proinflammatory T-cell responses to gut microbiota promote experimental autoimmune encephalomyelitis. Proc. Natl. Acad. Sci. USA *108* (*Suppl 1*), 4615–4622.

Lee, Y., Awasthi, A., Yosef, N., Quintana, F.J., Xiao, S., Peters, A., Wu, C., Kleinewietfeld, M., Kunder, S., Hafler, D.A., et al. (2012). Induction and molecular signature of pathogenic TH17 cells. Nat. Immunol. *13*, 991–999.

Li, B., and Dewey, C.N. (2011). RSEM: accurate transcript quantification from RNA-Seq data with or without a reference genome. BMC Bioinformatics *12*, 323.

Love, M.I., Huber, W., and Anders, S. (2014). Moderated estimation of fold change and dispersion for RNA-seq data with DESeq2. Genome Biol. *15*, 550.

Mangan, P.R., Harrington, L.E., O'Quinn, D.B., Helms, W.S., Bullard, D.C., Elson, C.O., Hatton, R.D., Wahl, S.M., Schoeb, T.R., and Weaver, C.T. (2006). Transforming growth factor-beta induces development of the T(H)17 lineage. Nature *441*, 231–234.

Mardinoglu, A., Shoaie, S., Bergentall, M., Ghaffari, P., Zhang, C., Larsson, E., Bäckhed, F., and Nielsen, J. (2015). The gut microbiota modulates host amino acid and glutathione metabolism in mice. Mol. Syst. Biol. *11*, 834.

Michalek, R.D., Gerriets, V.A., Jacobs, S.R., Macintyre, A.N., MacIver, N.J., Mason, E.F., Sullivan, S.A., Nichols, A.G., and Rathmell, J.C. (2011). Cutting edge: distinct glycolytic and lipid oxidative metabolic programs are essential for effector and regulatory $CD4^+$ T cell subsets. J. Immunol. *186*, 3299–3303.

Mishra, P., and Chan, D.C. (2016). Metabolic regulation of mitochondrial dynamics. J. Cell Biol. *212*, 379–387.

Murphy, C.T., Hall, L.J., Hurley, G., Quinlan, A., MacSharry, J., Shanahan, F., Nally, K., and Melgar, S. (2012). The sphingosine-1-phosphate analogue FTY720 impairs mucosal immunity and clearance of the enteric pathogen Citrobacter rodentium. Infect. Immun. *80*, 2712–2723.

Olenchock, B.A., Rathmell, J.C., and Vander Heiden, M.G. (2017). Biochemical underpinnings of immune cell metabolic phenotypes. Immunity *46*, 703–713.

Patel, D.D., and Kuchroo, V.K. (2015). Th17 cell pathway in human immunity: lessons from genetics and therapeutic interventions. Immunity *43*, 1040–1051.

Patil, K.R., and Nielsen, J. (2005). Uncovering transcriptional regulation of metabolism by using metabolic network topology. Proc. Natl. Acad. Sci. USA *102*, 2685–2689.

R Development Core Team (2015). R: A Language and Environment for Statistical Computing (Vienna, Austria: R Foundation for Statistical Computing).

Rambold, A.S., and Pearce, E.L. (2018). Mitochondrial dynamics at the interface of immune cell metabolism and function. Trends Immunol. *39*, 6–18.

Ritchie, M.E., Phipson, B., Wu, D., Hu, Y., Law, C.W., Shi, W., and Smyth, G.K. (2015). limma powers differential expression analyses for RNA-sequencing and microarray studies. Nucleic Acids Res. *43*, e47.

Sano, T., Huang, W., Hall, J.A., Yang, Y., Chen, A., Gavzy, S.J., Lee, J.Y., Ziel, J.W., Miraldi, E.R., Domingos, A.I., et al. (2015). An IL-23R/IL-22 circuit regulates epithelial serum amyloid A to promote local effector Th17 responses. Cell *163*, 381–393.

Schindelin, J., Arganda-Carreras, I., Frise, E., Kaynig, V., Longair, M., Pietzsch, T., Preibisch, S., Rueden, C., Saalfeld, S., Schmid, B., et al. (2012). Fiji: an open-source platform for biological-image analysis. Nat. Methods *9*, 676–682.

Schnupf, P., Gaboriau-Routhiau, V., and Cerf-Bensussan, N. (2013). Host interactions with Segmented Filamentous Bacteria: an unusual trade-off that drives the post-natal maturation of the gut immune system. Semin. Immunol. *25*, 342–351.

Schnupf, P., Gaboriau-Routhiau, V., Sansonetti, P.J., and Cerf-Bensussan, N. (2017). Segmented filamentous bacteria, Th17 inducers and helpers in a hostile world. Curr. Opin. Microbiol. *35*, 100–109.

Schreiber, H.A., Loschko, J., Karssemeijer, R.A., Escolano, A., Meredith, M.M., Mucida, D., Guermonprez, P., and Nussenzweig, M.C. (2013). Intestinal monocytes and macrophages are required for T cell polarization in response to Citrobacter rodentium. J. Exp. Med. *210*, 2025–2039.

Sherlock, J.P., Joyce-Shaikh, B., Turner, S.P., Chao, C.C., Sathe, M., Grein, J., Gorman, D.M., Bowman, E.P., McClanahan, T.K., Yearley, J.H., et al. (2012). IL-23 induces spondyloarthropathy by acting on ROR-γt^+ $CD3^+CD4^-CD8^-$ entheseal resident T cells. Nat. Med. *18*, 1069–1076.

Torchinsky, M.B., Garaude, J., Martin, A.P., and Blander, J.M. (2009). Innate immune recognition of infected apoptotic cells directs T(H)17 cell differentiation. Nature *458*, 78–82.

Umesaki, Y., Okada, Y., Matsumoto, S., Imaoka, A., and Setoyama, H. (1995). Segmented filamentous bacteria are indigenous intestinal bacteria that activate intraepithelial lymphocytes and induce MHC class II molecules and fucosyl asialo GM1 glycolipids on the small intestinal epithelial cells in the ex-germ-free mouse. Microbiol. Immunol. *39*, 555–562.

Valente, A.J., Maddalena, L.A., Robb, E.L., Moradi, F., and Stuart, J.A. (2017). A simple ImageJ macro tool for analyzing mitochondrial network morphology in mammalian cell culture. Acta Histochem. *119*, 315–326.

van der Windt, G.J., Everts, B., Chang, C.H., Curtis, J.D., Freitas, T.C., Amiel, E., Pearce, E.J., and Pearce, E.L. (2012). Mitochondrial respiratory capacity is a critical regulator of CD8+ T cell memory development. Immunity *36*, 68–78.

Väremo, L., Nielsen, J., and Nookaew, I. (2013). Enriching the gene set analysis of genome-wide data by incorporating directionality of gene expression and

combining statistical hypotheses and methods. Nucleic Acids Res. *41*, 4378–4391.

Wang, R., Dillon, C.P., Shi, L.Z., Milasta, S., Carter, R., Finkelstein, D., McCormick, L.L., Fitzgerald, P., Chi, H., Munger, J., and Green, D.R. (2011). The transcription factor Myc controls metabolic reprogramming upon T lymphocyte activation. Immunity *35*, 871–882.

Wang, C., Yosef, N., Gaublomme, J., Wu, C., Lee, Y., Clish, C.B., Kaminski, J., Xiao, S., Meyer Zu Horste, G., Pawlak, M., et al. (2015). CD5L/AIM regulates lipid biosynthesis and restrains Th17 cell pathogenicity. Cell *163*, 1413–1427.

Weaver, C.T., Elson, C.O., Fouser, L.A., and Kolls, J.K. (2013). The Th17 pathway and inflammatory diseases of the intestines, lungs, and skin. Annu. Rev. Pathol. *8*, 477–512.

Withers, D.R., Hepworth, M.R., Wang, X., Mackley, E.C., Halford, E.E., Dutton, E.E., Marriott, C.L., Brucklacher-Waldert, V., Veldhoen, M., Kelsen, J., et al. (2016). Transient inhibition of ROR-γt therapeutically limits intestinal inflammation by reducing TH17 cells and preserving group 3 innate lymphoid cells. Nat. Med. *22*, 319–323.

Witten, D.M. (2012). Classification and clustering of sequencing data using a Poisson model. Ann. Appl. Stat. *5*, 2493–2518.

Wu, H.J., Ivanov, I.I., Darce, J., Hattori, K., Shima, T., Umesaki, Y., Littman, D.R., Benoist, C., and Mathis, D. (2010). Gut-residing segmented filamentous bacteria drive autoimmune arthritis via T helper 17 cells. Immunity *32*, 815–827.

Wu, C., Yosef, N., Thalhamer, T., Zhu, C., Xiao, S., Kishi, Y., Regev, A., and Kuchroo, V.K. (2013). Induction of pathogenic TH17 cells by inducible salt-sensing kinase SGK1. Nature *496*, 513–517.

Yang, Y., Torchinsky, M.B., Gobert, M., Xiong, H., Xu, M., Linehan, J.L., Alonzo, F., Ng, C., Chen, A., Lin, X., et al. (2014). Focused specificity of intestinal TH17 cells towards commensal bacterial antigens. Nature *510*, 152–156.

Yu, X., Rollins, D., Ruhn, K.A., Stubblefield, J.J., Green, C.B., Kashiwada, M., Rothman, P.B., Takahashi, J.S., and Hooper, L.V. (2013). TH17 cell differentiation is regulated by the circadian clock. Science *342*, 727–730.

Zelante, T., Iannitti, R.G., Cunha, C., De Luca, A., Giovannini, G., Pieraccini, G., Zecchi, R., D'Angelo, C., Massi-Benedetti, C., Fallarino, F., et al. (2013). Tryptophan catabolites from microbiota engage aryl hydrocarbon receptor and balance mucosal reactivity via interleukin-22. Immunity *39*, 372–385.

STAR★METHODS

KEY RESOURCES TABLE

REAGENT or RESOURCE	SOURCE	IDENTIFIER
Antibodies		
anti-mouse CD16/CD32 purified	eBioscience	cat#16-0161-86; RRID: AB_468900
APC anti-mouse/human CD11b, clone M1/70	Biolegend	cat#101212; RRID: AB_312795
APC anti-mouse CD3ε, clone 145-2C11	Biolegend	cat#100312; RRID: AB_312677
APC anti-mouse Ly-6G/Ly-6C (Gr-1), clone RB6-8C5	Biolegend	cat#108412; RRID: AB_313377
APC anti-mouse CD19, clone 6D5	Biolegend	cat#115512; RRID: AB_313647
APC anti-mouse TCR-β chain, clone H57-597	Biolegend	cat#109212; RRID: AB_313435
APC anti-mouse TCRγδ, clone eBioGL3	eBioscience	cat#17-5711-82; RRID: AB_842757
PE-Cy7 anti-mouse CD90.2 (Thy1), clone 53-2.1	BD Bioscience	cat#561642; RRID: AB_10895975
Alexa Fluor 700 anti-Mouse CD45, clone 30-F11	BD Bioscience	cat#560510; RRID: AB_1645208
BV650 anti-mouse RORγt, clone Q31-378	BD Bioscience	cat#564722; RRID: AB_2738915
PE anti-mouse CD4, clone RMA-5	Biolegend	cat#100512; RRID: AB_312715
PercPcy5.5 anti-mouse TCR-β, clone H57-597	Biolegend	cat#109228; RRID: AB_1575173
APC anti-mouse IL-22, clone IL22JOP	eBioscience	cat#17-7222-82; RRID: AB_10597583
Pacific Blue anti-mouse IL-17A, clone TC11-18H10.1	Biolegend	cat#506918; RRID: AB_893544
PE-Cy7 anti-mouse IFN-γ, clone XMG1.2	Biolegend	cat#505826; RRID: AB_2295770
PE anti-mouse CD11c, clone N418	Biolegend	cat#117308; RRID: AB_313777
APC anti-mouse I-A/I-E (MHC class II), clone M5/114.15.2	Biolegend	cat#107614; RRID: AB_313329
Pacific blue anti-mouse CD45, clone 30-F11	Biolegend	cat#103126; RRID: AB_493535
Bacterial and Virus Strains		
C. rodentium infection strain ICC169	Dr.Gad Frankel, Imperial College London	N/A
His-EspA-expressing *E. coli*	Dougan Gordon, Sanger Institute	NA
Chemicals, Peptides, and Recombinant Proteins		
DPBS, no calcium, no magnesium	ThermoFisher	cat#14190250
Iscove's Modified Dulbecco's Medium	Sigma-Aldrich	cat#I3390
Fetal Bovine Serum	Sigma-Aldrich	cat#F7524
UltraPure 0.5M EDTA	ThermoFisher	cat#15575020
HEPES solution	Sigma-Aldrich	cat#H0887
penicillin/streptomycin	Sigma-Aldrich	cat#P7539
L-Glutamine (200 mM)	ThermoFisher	cat#25030149
Dithiothreitol (DTT)	ThermoFisher	cat#R0862
DNase I	Roche	cat#11284932001
Liberase TL	Sigma-Aldrich	cat#5401020001
Collagenase VIII	Sigma-Aldrich	cat#C2139
Collagenase D	Roche	cat#11088882001
Percoll	GE healthcare	cat#17-0891-01
phorbol-12-myristate-13 acetate (PMA)	Sigma-Aldrich	cat#P8139
ionomycin	Sigma-Aldrich	cat#I0634
Brefeldin A	Sigma-Aldrich	cat#B7651
Formaldehyde solution	Sigma-Aldrich	cat#F8775
1M Tris pH 8.0	Ambion	cat#AM9856
5M NaCl	Ambion	cat#AM9760G

(*Continued on next page*)

Continued

REAGENT or RESOURCE	SOURCE	IDENTIFIER
20% Sodium dodecyl sulfate (SDS)	Ambion	cat#AM9820
phenol:chloroform:isoamyl alcohol (25:24:1)	Ambion	cat#AM9730
3M Sodium acetate pH 5.5	ThermoFisher	cat#AM9740
Bovine serum albumin (BSA)	New England BioLabs	cat#B90005
FTY720	Cayman Chemical	cat#10006292
Dimethyl sulfoxide (DMSO)	Sigma-Aldrich	cat#D2650
Emulsion containing $MOG_{35–55}$ and killed *Mycobacterium tuberculosis*	Hooke Laboratories	cat#EK-0111
Pertussis toxin, *Bordetella pertussis*	Calbiochem	cat#516560
Propidium iodide	Sigma-Aldrich	cat#P4170
SFB peptide (NYU_003340) LVFDVQFSGAVPNKT	Crick Peptide Chemistry	N/A
Poly-D-Lysine	Sigma-Aldrich	cat#A-003-E
MitoTracker Red CMXRos	Thermofisher	cat#M7512
4’,6-diamidino-2-phenylindole (DAPI)	Sigma-Aldrich	cat#D9542
Alexa Fluor 488 Phalloidin	Thermofisher	cat#A12379
Critical Commercial Assays		
MILLIPLEX MAP mouse Th17 Magnetic Bead Panel kit	Merck	cat#MTH17-MAG-47K
LIVE/DEAD Fixable Near-IR Dead Cell	Thermofisher	cat#L10119
Fix/Perm buffer	eBioscience	cat#00-5523-00
SMART-Seq V4 ultra low input RNA kit	Takara	cat#634890
miRNeasy Micro kit	Qiagen	cat#217084
RiboPure RNA purification kit	Thermofisher	cat#AM1924
QIAquick PCR Purification kit	Qiagen	cat#28106
QuantiFAST SYBR Green	Qiagen	cat#204056
ThermoScript RT-PCR System kit	Thermofisher	cat#11146024
PCR Master Mix	Thermofisher	cat#4318157
EasySep Mouse $CD4^+$ T Cell Isolation kit	Stemcell technologies	cat#19752
Ni-NTA Spin kit	Qiagen	cat#31314
Seahorse XFp Cell Mito Stress Test kit	Agilent	cat#103010-100
Deposited Data		
RNA-seq data	This paper	GEO: GSE130302
Experimental Models: Organisms/Strains		
Il17a^{Cre}*R26R*^{eYFP}mice	Jackson Laboratory	Stock No. 016879
Oligonucleotides		
B2m (Mm00437762_m1)	Applied Biosystem	cat#4331182
Saa1 (Mm00656927_g1)	Applied Biosystem	cat#4331182
Fut2 (Mm00440152_s1)	Applied Biosystem	cat#4331182
Duoxa2 (Mm00470560_m1)	Applied Biosystem	cat#4331182
Il23a (Mm00518984_m1)	Applied Biosystem	cat#4331182
Cxcl1 (Mm00433859_m1)	Applied Biosystem	cat#4331182
Cxcl2 (Mm00436450_m1)	Applied Biosystem	cat#4331182
Il1b (Mm01336189_m1)	Applied Biosystem	cat#4331182
UniF340 (5′-ACTCCTACGGGAGGCAGCAGT-3′)	Thermofisher	N/A
UniR514 (5′-ATTACCGCGGCTGCTGGC-3′)	Thermofisher	N/A
SFB736F (5′-GACGCTGAGGCATGAGAGCAT-3′)	Thermofisher	N/A
SFB844R (5′-GACGGCACGGATTGTTATTCA-3′)	Thermofisher	N/A

(*Continued on next page*)

Continued		
REAGENT or RESOURCE	SOURCE	IDENTIFIER
Software and Algorithms		
GraphPad Prism 7.0c	GraphPad Software	https://www.graphpad.com/scientific-software/prism/
FlowJo LLC version 10.4.2	Becton Dickinson	https://www.flowjo.com/solutions/flowjo/downloads
Ingenuity Pathway Analysis (IPA)	QIAGEN	www.qiagen.com/ingenuity
Bowtie2	Langmead and Salzberg, 2012	N/A
RSEM	Li and Dewey, 2011	N/A
DESeq2 (version 1.16.1)	Love et al., 2014	N/A
R (version 3.4.1)	R Development Core Team, 2015	N/A
Limma package	Ritchie et al., 2015	N/A
DAVID analysis	Huang da et al., 2009	N/A
PIANO R package	Väremo et al., 2013	N/A
Mouse genome-scale metabolic model	Mardinoglu et al., 2015	N/A
WAVE (version 2.6)	Agilent	https://www.agilent.com/en/products/cell-analysis/cell-analysis-software/data-analysis/wave-desktop-2-6
ImageJ/Fiji	Schindelin et al., 2012	https://fiji.sc/#download
SVI Huygens	Scientific Volume Imaging (SVI)	https://svi.nl/Huygens-Essential
Mitochondrial Network Analysis (MiNA) macro for ImageJ/Fiji	Schindelin et al., 2012; Valente et al., 2017	N/A

LEAD CONTACT AND MATERIALS AVAILABILITY

Further information and requests for resources and reagents should be directed to and will be fulfilled by the Lead Contact (brigitta.stockinger@crick.ac.uk).

EXPERIMENTAL MODEL AND SUBJECT DETAILS

Mice

$Il17a^{Cre}R26R^{eYFP}$ mice (on a C57BL/6 background) were bred and maintained in individually ventilated cages at the Francis Crick Institute, under specific pathogen-free conditions according to the protocols approved by the UK Home Office and the ethics committee (AWERP) of the Francis Crick Institute. Sex-matched male and female littermates were randomly assigned to experimental groups in an age range of 4–10 weeks unless otherwise specified.

METHOD DETAILS

Colonization with Segmented Filamentous Bacteria

Mice in our colony were maintained SFB^- unless mentioned otherwise. To colonize mice with SFB, SFB^- mice were orally gavaged at weaning with SFB-containing feces collected from SFB^+ mice housed in our colony. Littermate controls were gavaged with SFB^- feces. SFB colonization status was routinely examined before and after SFB gavage by quantitative PCR.

Quantitative PCR for Segmented Filamentous Bacteria

Flash-frozen stools were transferred in a Precellys's soil grinding tube (Stretton Scientific) with 500 μl of Tris-EDTA buffer containing 200 mM Tris (pH 8.0), 20 mM EDTA, 200 mM NaCl; 210 μl of sodium dodecyl sulfate (SDS 20%) and 500 μl of phenol:chloroform:isoamyl alcohol (25:24:1) (all from Ambion, Life technologies). Samples were homogenized with a Precellys 24 homogenizer (Bertin Technologies). The aqueous phase was removed after centrifugation and washed with isopropanol and sodium acetate (pH 5.5) (all from ThermoFisher). Samples were incubated at −20°C for 1 h, washed with 70% ethanol (ThermoFisher), resuspended in TE buffer containing 10 mM Tris (pH 8.0) and 1 mM EDTA and incubated at 50°C for 20 min. DNA was further purified using QIAquick PCR Purification kit (Quiagen), quantified with Nanodrop Lite Spectrophotometer (Thermofisher) and diluted to equal concentrations. Quantitative PCR was performed with a QuantStudio Real-Time PCR (Thermofisher) using QuantiFAST SYBR Green (Quiagen), 1 μg/ul BSA (New England BioLabs) and the following primers for Eubacteria (UniF340 and UniR514) and SFB (SFB736F and SFB844R):

UniF340: (5′ACTCCTACGGGAGGCAGCAGT-3′)
UniR514: (5′ATTACCGCGGCTGCTGGC-3′)
SFB736F: (5′-GACGCTGAGGCATGAGAGCAT-3′)
SFB844R: (5′GACGGCACGGATTGTTATTCA-3′)

For Eubacterial primers, initial 95°C for 5 min, followed by 45 cycles of 95°C for 10 seconds and 63°C for 45 seconds. For SFB primers initial 95°C for 5 min, followed by 45 cycles of 95°C for 10 seconds and 58°C for 45 seconds. SFB abundance was calculated by the threshold cycle (ΔCt) method and normalized to Eubacteria.

Quantitative PCR for intestinal tissue

RNA was isolated from colon or small intestine using the RiboPure RNA purification kit, according to the manufacturer's instructions. One microgram total RNA was reverse-transcribed using the ThermoScript RT-PCR System kit (Applied biosystem) accordingly to manufacturer's instructions. The cDNA served as a template for the amplification of genes of interest and the housekeeping gene (beta-2-microglobulin, β2m) by real-time quantitative PCR, using TaqMan Gene Expression Assays (Applied Biosystems), universal PCR Master Mix (Applied Biosystems) and the ABI-PRISM 7900 sequence detection system (Applied Biosystems). mRNA expression was determined using the ΔCt method, relatively to *B2m* gene expression.

Infection with Citrobacter rodentium

For *C. rodentium* infection, a single colony of strain DBS100 (ATCC 51459; American Type Culture Collection) was transferred to Luria-Bertani (LB) broth and grown to log phase, followed by centrifugation and resuspension in PBS. Mice were orally gavaged with 200 μl of PBS containing 2×10^9 *C. rodentium* CFU. To determine bacterial load, tissue pieces were weighed and homogenized in sterile PBS and serial dilutions were plated onto Brilliance *E. coli*/coliform Selective Agar (Fisher Scientific) or LB agar plates (liver) for measurement of colony-forming units (CFU). For the administration of FTY720, six days prior the infection with *C. rodentium* or PBS gavage, mice were intra-peritoneally injected with 3mg/kg FTY720 (Cayman Chemical) resuspended in PBS + 5% DMSO or control vehicle, every day for six days. After *C. rodentium* infection or PBS gavage, the administration of the drug continued every other day until the end of the experiment.

Cell isolation

Colon and small intestine were cut open longitudinally and incubated in wash buffer (IMDM 1%FCS, 5 mM EDTA, 10 mM HEPES, penicillin/streptomycin, and 2 mM DTT) for 20 min at 37°C with 200 r.p.m. shaking. Colon tissue was collected, cut into small pieces and incubated in digestion buffer (IMDM supplemented with 1% FCS, 10 mM HEPES, penicillin/streptomycin, 50 μg/ml DNase I (Roche)) containing 0.4cmg/ml Liberase TL (Roche) for 30 cmin at 37c°C with 200 r.p.m. shaking. Small intestinal tissue was collected, cut into small pieces and incubated in digestion buffer (IMDM supplemented with 1% FCS, 10 mM HEPES, penicillin/streptomycin, 50 μg/ml DNase I (Roche)) containing 1 mg/ml collagenase VIII (Sigma) for 10 min at 37°C with 200 r.p.m. shaking. Single-cell suspensions from colon and small intestine were further subjected to 40% Percoll (Amersham) density gradient centrifugation to remove debris. Spinal cord from mice with a clinical score of 3 after immunization with MOG-CFA was mashed through 70-μm mesh filter and subjected to 40% Percoll density gradient centrifugation. Spleen was mashed through 70-μm mesh filter and red blood cells lysed with ACK lysing buffer, containing 0.15 M NH_4Cl, 1 mM $KHCO_3$ and 0.1 mM Na_2EDTA with adjusted pH 7.2–7.4.

Flow cytometry and cell sorting

Single cell suspensions were prepared as described from the indicated organ and incubated with anti-CD16/32 (eBioscience) and fixable live/dead cell dye (ThermoFisher). Cell suspensions for Th17 staining were incubated with antibodies against CD45 (BD Biosciences), CD4 and TCR-β (Biolegend) whereas cell suspensions for ILC3 staining were incubated with antibodies against CD11b, CD3, TCRγδ, Gr1, CD19, TCR-β (all BioLegend) and Thy1.2, CD45 (all BD Biosciences). For intracellular staining, single-cell suspensions were re-stimulated for 2 h in the presence of 1 ng/ml phorbol-12-myristate-13-acetate (PMA), 1μg/ml ionomycin and 10 μg/ml Brefeldin A (all Sigma), washed and stained for surface markers as described above. Cells were then fixed in eBioscence Fix/Perm buffer or 3.7% formaldehyde (for preservation of eYFP fluorescence) for 30 or 10 min on ice, respectively, followed by permeabilization in eBioscience permeabilization buffer for 30 min in the presence of antibodies against IL-22 (eBioscience), IFN-γ and IL17-A (BioLegend) for Th17 or RORγt (BD Bioscences) for ILC3. Samples were FSC-A/SSC-A gated to exclude debris, SSC-W/SSC-A and FSC-W/FSC-A gated to select single cells and gated to exclude live/dead-dye+ dead cells. Th17 cells were gated as $CD45^+CD4^+TCR\text{-}\beta^+eYFP^+$. ILC3 cells were gated as $CD45^+CD11b^-CD3^-TCR\gamma\delta^-Gr1^-CD19^-TCR\text{-}\beta^-Thy1.2^+ROR\gamma t^+$. Samples were run on a BD Fortessa X20 and analysis was performed with FlowJo v10 (Tree Star) software. For Th17 cell sorting, single-cell suspensions from the spleen were enriched for CD4 T cells with EasySep Mouse $CD4^+$ T Cell Isolation kit according to manufacturer's instructions, whereas suspensions from the intestine were FACS-sorted without any prior enrichment. Cell suspensions were stained with propidium iodide (Sigma) to exclude dead cells and with antibodies against CD45, CD4, and TCR-β (all from Biolegend) and Th17 cells sorted as live $CD45^+CD4^+TCR\text{-}\beta^+eYFP^+$ cells. For dendritic cell sorting, spleens from RAG-$1^{-/-}$ mice were incubated in digestion buffer (IMDM supplemented with 1% FCS, 10 mM HEPES, penicillin/streptomycin) containing 1 mg/mL Collagenase D and 20 μg/mL DNase I (all from Roche) for 1 h at 37°C. Cell suspensions were then mashed through 70-μm mesh filter, lysed for red blood cells and stained with propidium iodide and antibodies against CD45, CD11c,

and MHC-class II (all from Biolegend). Dendritic cells were sorted as live $CD45^{+}CD11c^{+}$MHC-class II^{high} cells. Cell sorting were performed using a MoFlo XDP (Beckman Coulter).

Th17 cell culture

Th17 cells were sorted as described and plated at 10^4 cells per well. At 24 h cytokines were measured in the supernatant by MILLIPLEX® MAP mouse Th17 Magnetic Bead Panel kit (Merck). For co-culture experiments Th17 cells were plated at 10^4 cells per well with dendritic cells at 3:1 ratio, together with 2.5 μM SFB peptide or 1 μg/mL EspA.

Recombinant proteins

SFB peptide derived from SFBNYU_003340 (LVFDVQFSGAVPNKT) (Yang et al., 2014) was generated by the Crick Peptide Chemistry platform. EspA was purified from His-EspA-expressing *E. coli* (Knutton et al., 1998), kindly provided by Dougan Gordon (Sanger Institute), using Ni-NTA Spin kit (Qiagen) according to manufacturer's instructions.

EAE induction

Mice were injected subcutaneously at two sites with 100μl of an emulsion containing 250 μg MOG peptide (amino acids 35–55) and 250 μg killed *Mycobacterium tuberculosis* (strain H37Ra) from Hooke Labs (Hooke Laboratories). Mice received 200 ng *Bordetella pertussis* (Calbiochem) intraperitoneally on the day of immunization and 2 d later. Clinical assessment of EAE was done daily and clinical scores were assigned according to the following criteria: 0, unaffected; 1, flaccid tail; 2, impaired righting reflex and/or gait; 3, partial hindlimb paralysis; 4, total hind limb paralysis. All mice with a clinical score of 4 were killed.

Colon explant cultures

Intestinal tissue pieces (0.5–1 cm length) were cultured for 24ch in complete IMDM medium. Cytokines were measured in the supernatant by MILLIPLEX® MAP mouse Th17 Magnetic Bead Panel kit (Merck) and concentrations were normalized to the weight of the explants.

RNA-sequencing

Single cell suspensions from small intestine and colon were generated, stained and sorted as described above to purify Th17 cells. Each replicate was pooled from three to six mice and three biological replicates were collected for each group, with the exception of *C. rodentium*-induced colonic Th17 cells, with five biological replicates. RNA was isolated with miRNeasy Micro kit (Quiagen) according to the manufacturer's protocol. Amplified cDNA was prepared using the SMART-Seq V4 ultra low input RNA kit (Takara), according to the manufacturer's instructions. Sequencing libraries were generated using the Ovation Ultralow V2 kit (NuGEN) according to manufacturer's instruction. Libraries were assessed using the TapeStation 2200 (Agilent Technologies) and sequenced by paired-end 100 base pair reads using the Illumina HiSeq platform. Reads were aligned to the GRCm38 Ensembl Release 86 mouse genome using Bowtie2 (Langmead and Salzberg, 2012) and quantified using RSEM (Li and Dewey, 2011). Differential genes were found using DESeq2 (Love et al., 2014) (version 1.16.1) in R (R Development Core Team, 2015) (version 3.4.1), using a two-factor model with interactions to account for difference between tissues, differences between treatments, and tissue-specific treatment effect. We used a false discovery threshold of 0.05 on the resulting Wald statistic. All four experimental conditions were used to estimate per-gene dispersion values. For the heat map of condition similarity (Figure 5A), each experimental group was summarized as the sum across replicates of the raw counts. The Poisson distance (Witten, 2012) between pairs of experimental groups was taken to assess how similar the conditions are using complete hierarchical clustering. For the scatter plot of differentially expressed genes (Figure 5C), low normalized read count (average across conditions less than 4) were removed to ensure the trend was not driven by noise: omitting this filtering showed no qualitative difference. For the scatter plot in Fig.S5D we compared our differentially expressed gene list generated from colonic SFB- versus *C. rodentium*-elicited Th17 cells to a publicly available expression dataset (GSE23505). Firstly, we identified the list of differentially expressed genes using the Limma package in R and selected genes that were statistically significant (p value < 0.001) in the published dataset. We then compared the generated gene list to the differentially expressed genes in our dataset (adjusted p value < 0.05). For canonical pathway analysis (Fig.5D), differentially expressed genes between SFB- and *C.-rodentium*-elicited colonic Th17 cells with adjusted p value < 0.05 and fold change > 1.5 were input in Ingenuity Pathway Analysis (IPA, QIAGEN Redwood City, www.qiagen.com/ingenuity). Enriched KEGG metabolic pathways (Fig.6A) and reporter metabolites (Fig.6B) (Patil and Nielsen, 2005) were identified from differentially expressed genes in SFB- versus *C. rodentium*-elicited colonic Th17 cells (adjusted p value < 0.05). Specifically, we used DAVID analysis (Huang da et al., 2009) to identify KEGG enriched pathways from up-regulated genes and PIANO R package (Väremo et al., 2013) to identify reporter metabolites from differentially expressed genes. Enzymes, metabolites and their topological relations used for the metabolic analysis were obtained from a mouse genome-scale metabolic model (Mardinoglu et al., 2015). In KEGG metabolic pathway analysis, we excluded metabolic pathways not originated from the host, such as biosynthesis of antibiotics, drug metabolism by cytochrome P450 and metabolism of xenobiotics by cytochrome P450. Consistently, in the reporter metabolite analysis, we excluded metabolites originating from these pathways, together with artificial reactions inferred on the genome-scale metabolic model and focused on up-regulated metabolites. A selection of reporter metabolites is displayed in Fig.6B, the complete list of results can be found in Table S1.

Metabolic assays

After FACS-sorting, Th17 cells (7×10^4 per well) were seeded onto standard Seahorse 8-well plates coated with Poly-D-Lysine (50 μg/mL, Sigma) and assayed on a Seahorse XFp Analyzer (Agilent Technologies, Santa Clara, USA). The plates were centrifuged at 300g for 5 min and kept at 37°C for 30–60 min. Oxygen consumption rates (OCR) and extracellular acidification rates (ECAR) were measured in XF media with pH adjusted to 7.4. To investigate mitochondrial respiration and energetic phenotypes Seahorse XFp Cell Mito Stress Test kit (Agilent Technologies) was used. The injection strategy was as follow, first: oligomycin (1 μM at final concentration), second: carbonyl cyanide 4-(trifluoromethoxy) phenylhydrazone (FCCP) (1 μM at final concentration), and third: rotenone and antimycin A (0.5 μM at final concentration). The WAVE software (version 2.6) was applied for further data analysis.

Confocal microscopy

After FACS-sorting, Th17 cells were cultured in μ-Slide 8 well glass bottom slides (ibidi GmbH) and incubated for 30 min at 37°C with MitoTracker™ Red CMXRos (100 nM, ThermoFisher). After two rinses with PBS, cells were fixed with paraformaldehyde 3.7% for 15 min. DAPI (1 μg/mL, Sigma) and Alexa Fluor™ 488 Phalloidin (150 nM, ThermoFisher) were used for nuclear and actin staining, respectively.

Z-stack images were acquired using a Leica TCS SP8 (Leica Microsystems, Germany) confocal microscope and spatial deconvolution and 3D surface-rendered images were carried out with Huygens Software (SVI, The Netherlands). Mitochondrial morphology was analysed using the macro MiNa in Fiji software (Schindelin et al., 2012; Valente et al., 2017).

QUANTIFICATION AND STATISTICAL ANALYSIS

Details of the statistical tests applied to replicates of datasets shown in Figures can be found in the corresponding Figure legends. All data points and ‘‘n’’ values reflect biological replicates (e.g., mice), except in Figures 2E, 3B, and 3C where they represent technical replicates. Statistical analyses were performed using GraphPad Prism 10 software.

DATA AND CODE AVAILABILITY

The accession number for the mRNA sequencing data reported in this paper is GenBank: GSE130302.

Immunity, Volume *51*

Supplemental Information

The Intestine Harbors Functionally Distinct Homeostatic Tissue-Resident and Inflammatory Th17 Cells

Sara Omenetti, Claudio Bussi, Amina Metidji, Andrea Iseppon, Sunjae Lee, Mauro Tolaini, Ying Li, Gavin Kelly, Probir Chakravarty, Saeed Shoaie, Maximiliano G. Gutierrez, and Brigitta Stockinger

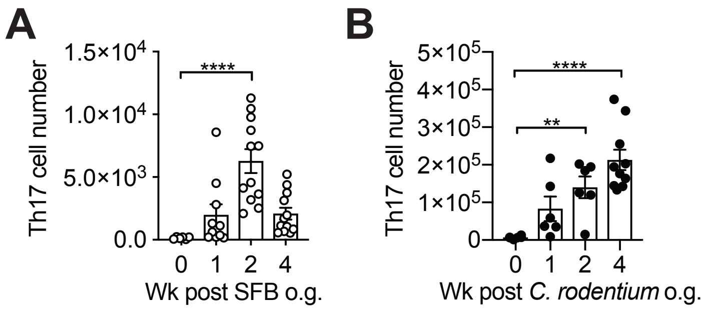

Figure S1. Th17 cell induction by SFB in the colon and C. rodentium in the small intestine, related to Figure 1.

Absolute numbers of Th17 cells in the colon of mice colonised with SFB (A) and the small intestine of mice infected with C. rodentium (B) at 0 (n=6), 1 (n=10-6), 2 (n=12-6) and 4 (n=12-10) weeks post-gavage. In the graphs, bars show the mean and each symbol represent an individual mouse from two pooled independent experiments. $^{**}p < 0.01$, $^{****}p < 0.0001$ by one-way ANOVA with Dunnett's post-test.

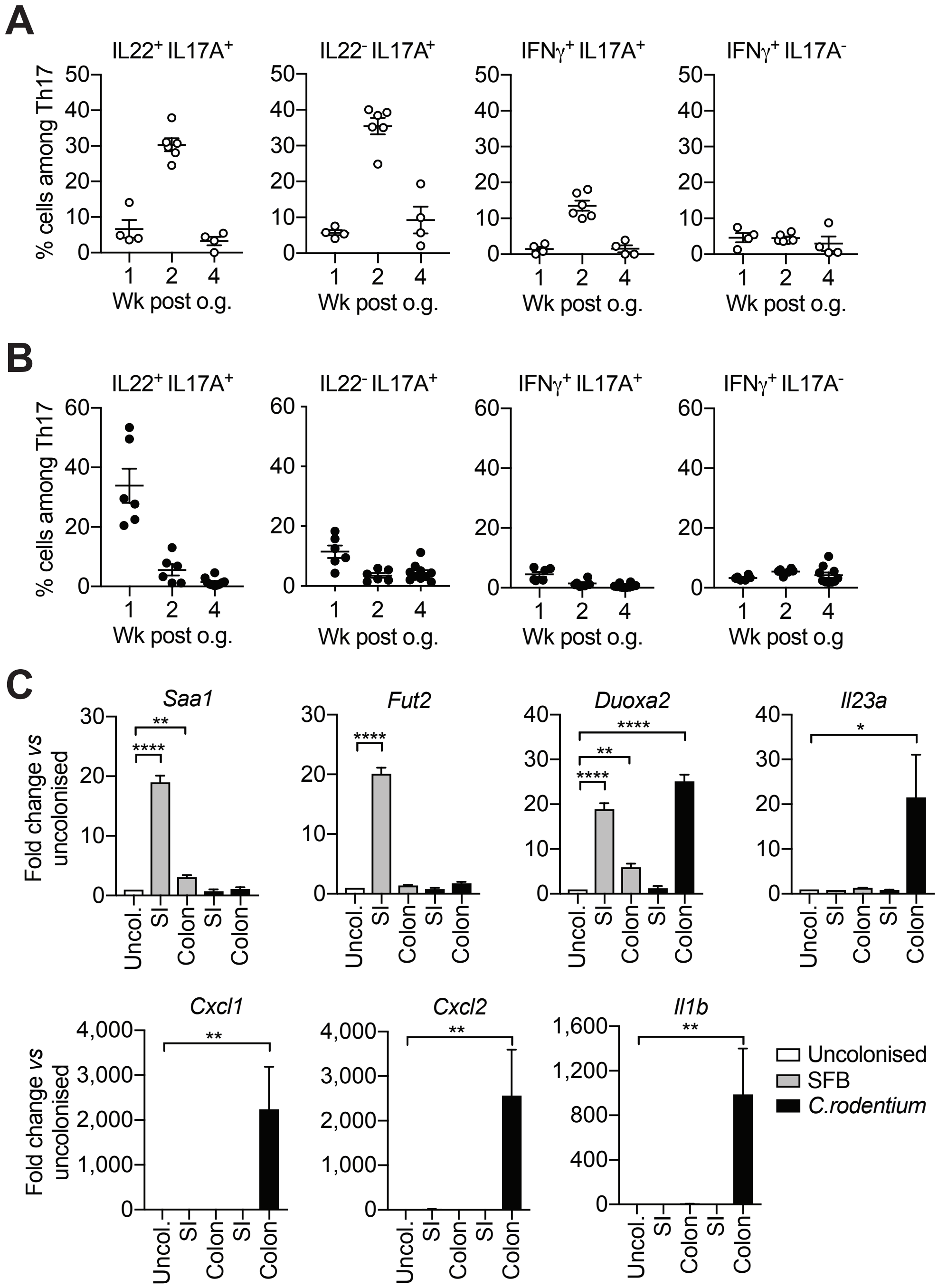

Figure S2. Cytokine production by SFB-elicited Th17 cells in the colon, related to Figure 2. Quantification of intracellular staining for IL-17A and IL-22 or IFN-γ in Th17 cells from the colon of mice colonised with SFB (A) and the small intestine of mice infected with C. rodentium (B) at 1 (n=4 and 6), 2 (n=6) and 4 (n=4 and 10) weeks post-gavage. Lamina propria cells were isolated at the indicated time-point, re-stimulated with PMA and Ionomycin and Brefeldin A for 2 hours and analysed by FACS. Bars show the mean ± s.e.m. for the indicated populations and each symbol represents an individual mouse from two pooled independent experiments. (C) Fold change induction of listed genes quantified by qPCR in the SI and colon of mice colonised with SFB (n=3 and n=4) or infected with C. rodentium (n=5), 1 week post-gavage. Fold change is calculated on the matching uncolonised organ (uncol.) (n=6) and gene expression normalised against ß-2-microglobulin. Bars show the mean + s.e.m. from two pooled independent experiments. *$p < 0.05$, **$p < 0.01$, ****$p < 0.0001$ by one-way ANOVA with Dunnett's post-test.

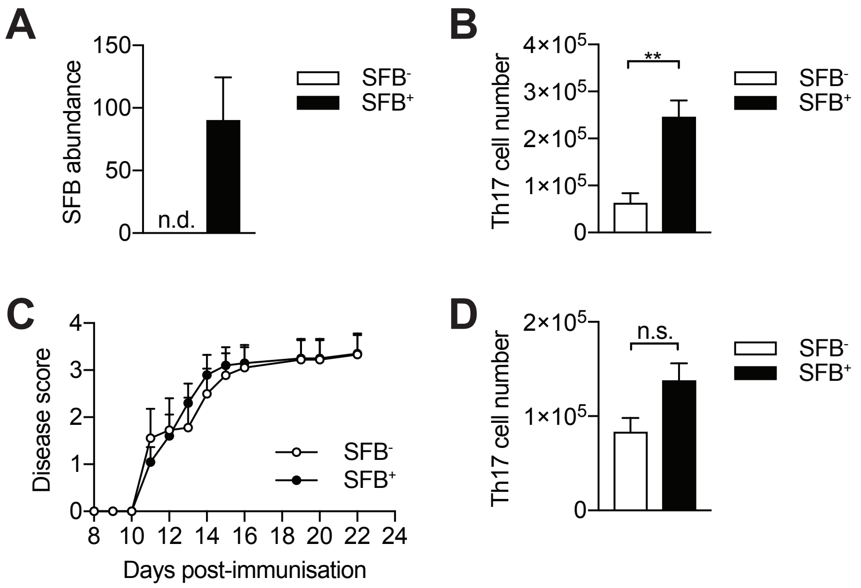

Figure S3. SFB does not impact the course of EAE, related to Figure 3.
(A) Relative abundance of Segmented Filamentous bacteria (SFB) in the faeces of mice reconstituted with SFB$^-$ (n=4) or SFB$^+$ (n=6) faeces at 12 days upon EAE immunisation. SFB genomic 16s was quantified in the faeces by qPCR analysis. Abundance of SFB was normalised to Eubacteria. (B) Absolute numbers of Th17 cells in the small intestine (B) or spinal cord (D) of SFB$^-$ and SFB$^+$ mice (n=4) at 12 days upon EAE immunisation. (C) Clinical scores of SFB$^-$ (n=10) and SFB$^+$ mice (n=10) mice upon EAE immunisation. Results are pooled from two independent experiments. Data are shown as mean + s.e.m. **$p < 0.01$ by Student t-test. n.s., not significant; n.d., not detected.

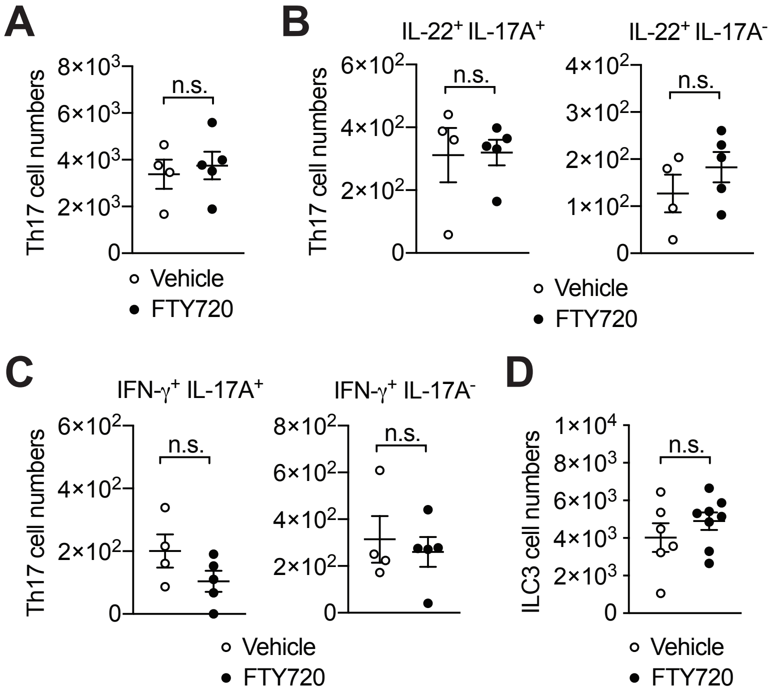

Figure S4. FTY720 treatment does not affect numbers of SFB-elicited Th17 cells and C. rodentium-elicited ILC3 cells, related to Figure 4.

Mice were orally gavaged with SFB+ faeces 4 weeks prior the gavage with PBS (A, B, C) or C.rodentium (D) (at day 0). Six days prior the PBS or C.rodentium gavage, mice were intra-peritoneally injected with FTY720 (3mg/kg) or control vehicle every day. FTY720 and control vehicle administration continued after the PBS or C. rodentium gavage every other day until the end of the experiment. (A) Absolute numbers of colonic Th17 cells (n=4 and n=5). Quantification of intracellular staining in colonic Th17 cells (n=4 and n=5) for IL-17A and IL-22 (B) or IFN-γ (C). (D) Absolute numbers of colonic ILC3 cells (n=6 and n=8). Bars show mean ± s.e.m. and each symbol represent an individual mouse. Results are representative of two independent experiments. n.s., not significant by Student t-test.

A

C

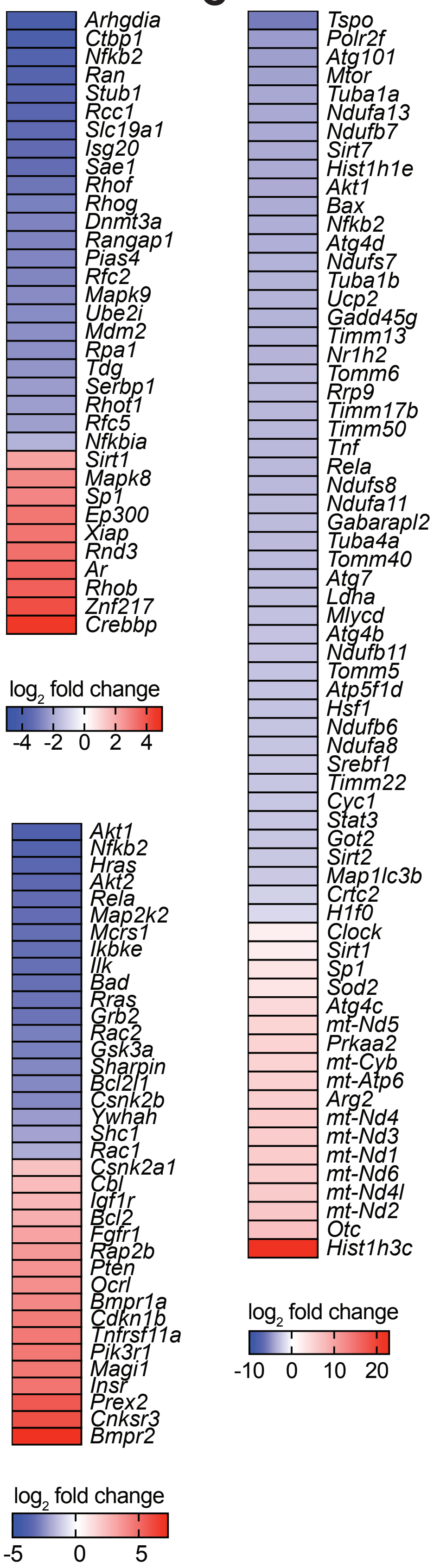

D

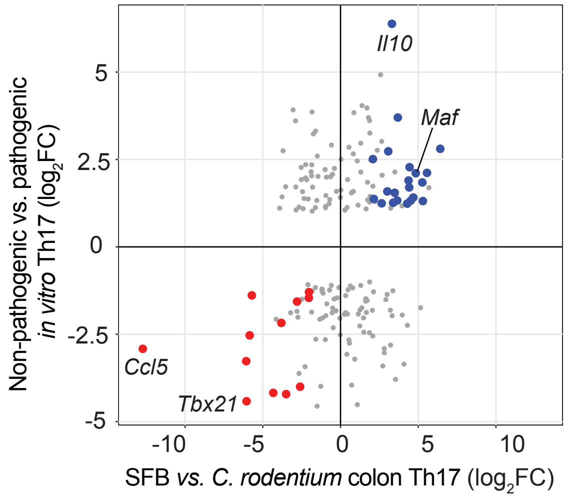

E

	SFB *vs. C.rodentium* colonic Th17	Non-pathogenic *vs.* pathogenic Th17
Lgr4	6.4	2.8
Nhsl1	5.6	2.1
Ptgfrn	5.3	1.3
Dfna5	5.3	1.8
Maf	4.8	2.1
Sgce	4.7	1.4
Pik3r1	4.5	1.3
Acvr2a	4.4	2.3
Alpk2	4.4	1.7
Sgip1	4.4	1.9
Mob3b	4.3	1.2
Gcnt1	3.7	3.7
Sfmbt2	3.6	1.3
Trio	3.5	1.5
Dcbld2	3.4	1.3
Il10	3.3	6.4
Prnp	3.1	2.7
Tmigd1	3.0	1.6
Skil	2.6	1.2
Pitpnc1	2.1	1.4
Acpp	2.1	2.5

F

	SFB *vs. C.rodentium* colonic Th17	Non-pathogenic *vs.* pathogenic Th17
Ccl5	-12.7	-2.9
Ifitm1	-6.1	-3.3
Tbx21	-6.0	-4.4
Ermn	-5.8	-2.5
Sell	-5.7	-1.4
Nkg7	-4.3	-4.2
Serpine2	-3.8	-2.2
Ifitm3	-3.5	-4.2
Lrmp	-2.8	-1.6
Cxcr3	-2.6	-4.0
Mbnl3	-2.0	-1.5
Uba7	-2.0	-1.3

Figure S5. Gene expression of pathways activated in SFB-induced colonic Th17 cells and shared gene expression with in vitro generated Th17 cells, related to Figure 5.
Expression (log2-fold change) of genes identified by ingenuity pathway analysis (IPA) in sumoylation (A), PTEN (B) and sirtuin (C) pathways in colonic SFB- vs. C.rodentium-induced Th17 cells. (D) Scatter plot of genes identified from in vitro generated non-pathogenic vs. pathogenic Th17 cells (Ghoreschi et al, 2010) ($p < 0.001$) detected in SFB or C. rodentium colon Th17 cells. Genes in blue (listed in E) and in red (listed in F) are significantly different in SFB vs. C. rodentium colon Th17 cells (adj $p < 0.05$) and follow the same direction of the published dataset. Values in E and F are $\log_2$ fold change of genes in the indicated comparison.
